# Grimace scale, burrowing, and nest building for the assessment of post-surgical pain in mice and rats—A systematic review

**DOI:** 10.3389/fvets.2022.930005

**Published:** 2022-10-06

**Authors:** Katharina Aulehner, Cathalijn Leenaars, Verena Buchecker, Helen Stirling, Katharina Schönhoff, Hannah King, Christine Häger, Ines Koska, Paulin Jirkof, André Bleich, Marion Bankstahl, Heidrun Potschka

**Affiliations:** ^1^Institute of Pharmacology, Toxicology and Pharmacy, Ludwig-Maximilians-University, Munich, Germany; ^2^Institute for Laboratory Animal Science, Hannover Medical School, Hanover, Germany; ^3^Office for Animal Welfare and 3Rs, University of Zurich, Zurich, Switzerland

**Keywords:** pain assessment, RGS, MGS, facial expression, home cage behavior, rodents

## Abstract

Several studies suggested an informative value of behavioral and grimace scale parameters for the detection of pain. However, the robustness and reliability of the parameters as well as the current extent of implementation are still largely unknown. In this study, we aimed to systematically analyze the current evidence-base of grimace scale, burrowing, and nest building for the assessment of post-surgical pain in mice and rats. The following platforms were searched for relevant articles: PubMed, Embase *via* Ovid, and Web of Science. Only full peer-reviewed studies that describe the grimace scale, burrowing, and/or nest building as pain parameters in the post-surgical phase in mice and/or rats were included. Information about the study design, animal characteristics, intervention characteristics, and outcome measures was extracted from identified publications. In total, 74 papers were included in this review. The majority of studies have been conducted in young adult C57BL/6J mice and Sprague Dawley and Wistar rats. While there is an apparent lack of information about young animals, some studies that analyzed the grimace scale in aged rats were identified. The majority of studies focused on laparotomy-associated pain. Only limited information is available about other types of surgical interventions. While an impact of surgery and an influence of analgesia were rather consistently reported in studies focusing on grimace scales, the number of studies that assessed respective effects was rather low for nest building and burrowing. Moreover, controversial findings were evident for the impact of analgesics on post-surgical nest building activity. Regarding analgesia, a monotherapeutic approach was identified in the vast majority of studies with non-steroidal anti-inflammatory (NSAID) drugs and opioids being most commonly used. In conclusion, most evidence exists for grimace scales, which were more frequently used to assess post-surgical pain in rodents than the other behavioral parameters. However, our findings also point to relevant knowledge gaps concerning the post-surgical application in different strains, age levels, and following different surgical procedures. Future efforts are also necessary to directly compare the sensitivity and robustness of different readout parameters applied for the assessment of nest building and burrowing activities.

## Introduction

The detection and scoring of post-surgical pain in laboratory animals are of particular relevance for several reasons. First, they are a prerequisite for severity assessment in studies with surgical interventions, which provides the basis for ethical justification and consideration prior to the conduct of the study and for an evidence-based retrospective evaluation (1, 2). Second, reliable pain assessment is required for decisions about the necessity of analgesia and the choice of an appropriate analgetic regimen. In animals receiving analgesic drugs, scoring pain enables controlling for therapeutic efficacy, thereby providing a basis for additional rescue analgesia in individual animals and for refinement measures including an adjustment of the pain management. In this context, rescue analgesia refers to the treatment of breakthrough pain by administering additional analgesics, either increasing the dose, using a different route of administration, or adding a more potent analgesic.

Moreover, pain assessment is a prerequisite for the application of humane endpoints in various animal models. Finally, it should be considered that different degrees of uncontrolled pain contribute to the variance of data obtained in animal models, thereby increasing the number of animals needed. Resultantly, uncontrolled or insufficiently controlled pain can significantly affect various readout parameters and can therefore restrict study quality (3–5). In this context, manifold effects need to be considered when including effects on the neuroendocrine, immune, cardiovascular, respiratory, autonomous, and central nervous systems.

Thus, the precise detection and scoring of pain are crucial prerequisites for the consequent application of reduction and refinement concepts in laboratory animal science, as defined in the 3R principles (6).

Unfortunately, despite strong efforts to develop and validate methods and techniques for pain detection and scoring, a highly reliable and reproducible pain assessment strategy in daily laboratory practice is yet to be achieved. This is related to various challenges that one has to face when it comes to pain assessment in different animal species. These challenges start with the distinction between nociception and pain. Whereas nociception describes signal transduction from the specialized sensory cellular nociceptors to the central nervous system, pain in animals is considered as an “aversive, sensory experience representing awareness by the animal of damage or threat to the integrity of its tissues (note that there might not be any damage). It changes the animal's physiology and behavior to reduce or avoid the damage, to reduce the likelihood of its recurrence and to promote recovery” (7). When evaluating biochemical and physiological parameters in animals, it needs to be considered that some of the parameters among others, including substance P, cardiovascular, and respiratory parameters, might be modulated by nociception even when the conscious experience of pain is prevented by pharmacological measures.

When it comes to pain assessment, another major challenge is related to the fact that prey animals should rather avoid displaying symptoms of pain and suffering (8–10). In laboratory animals, this prey animal effect has been demonstrated, for example, in mice, rats, rabbits, and sheep (11, 12). Thus, the observer's presence may exert an influence and, consequently, familiarity with the observer is considered advantageous.

Agreement exists that composite measure schemes combining various physiological, endocrine, and behavioral parameters need to be applied to assess pain in laboratory mice and rats as reliably as possible (10). Several reviews summarized and discussed the informative value of various pain assessment methods (10, 13–18). For instance, as highlighted by Turner et al. (10) in their wide-ranging review, different behavioral parameters have been implemented for the analysis of pain in laboratory rodents. Ethograms have, for instance, been described and validated for mice and rats that capture information about the occurrence of pain-associated patterns, such as writhing and back-arching, and about the reduction or loss of normal species-specific behavioral patterns such as grooming and rearing (19–26). Behavioral patterns that can be reduced as a consequence of pain also include the interaction with nesting or burrowing material. Both, nest building and burrowing are evolutionary preserved activities in rodents that are considered non-essential in the laboratory animal facility environment (8, 9). The particular sensitivity of nest building and burrowing activity to pain, including post-surgical pain, has been reported in various studies (8, 27–31). In this context, it should also be considered that several studies provided evidence that mice and rats have various species-specific behavioral needs, and when these are not fulfilled, abnormal behavioral patterns can occur (32). Along this line, pain, sickness, and compromised welfare can exert effects on species-specific behavioral patterns.

As another valuable parameter, facial expression patterns reflecting the experience of pain have been reported across species boundaries (33–39). While early descriptions by Darwin already suggested parallels in facial expressions reflecting emotions in different animal species and humans, Langford et al. (33) were the first group to systematically study facial expressions as a measure of pain. Their groundbreaking work focused on the Mouse Grimace Scale (MGS) and its thorough validation in a variety of pain models (33). Using grimace scales, deviations from the physiological state can be examined based on the facial expressions. The grimace scale for mice comprises five action units (AUs): orbital tightening, nose bulge, cheek bulge, ear position, and whisker change (33). Subsequently, grimace scales have been developed and assessed in different species including rats (34–38, 40).

Regardless of the pain parameter, one needs to consider the pronounced influence of numerous variables such as genetics, age, sex, environmental factors, social interaction, and prior experience that can influence pain assessment (10, 14, 16, 41–44). Despite the fact that extensive narrative reviews have been published, summarizing available information about the value, practical use, and limitations of grimace scales, nest building performance, and burrowing activity as parameters for the assessment of pain (9, 41), there is still a knowledge gap concerning the implementation of these methods for the assessment of post-surgical pain. To our knowledge, more systematic approaches have so far only been used in reviews focused on MGS.

A scoping review by Whittaker et al. examined the MGS in different types of pain (e.g., visceral pain after injection of Freund's adjuvant), indicating a wide application of the MGS in different animal models (45). A recent scoping review focusing on the grimace scales in non-human mammals has already intensely studied the level of evidence for measurement properties of various grimace scales reporting a high level of evidence for MGS and RGS (46). Our analysis is more specifically focused on the application of post-operative pain induced by different types of surgeries under general anesthesia in mice and rats, including the extraction of information about anesthesia and perioperative analgesia.

Taken together, there are remaining knowledge gaps concerning the implementation of grimace scales for post-surgical pain assessment and obvious knowledge gaps concerning the post-surgical implementation of nest building and burrowing assessment.

Therefore, we completed a systematic review exploring the available literature about the application of grimace scales and the assessment of nest building and burrowing activity in the context of surgical interventions and associated pain.

## Materials and methods

The systematic review protocol was registered before starting the formal screening of papers with the Systematic Review Facility (SyRF) for preclinical studies on 26 March 2020 and is available from https://syrf.org.uk/protocols and in Supplementary Methods 1. We used the Systematic Review Center for Laboratory animal Experimentation's (SYRCLE) protocol template version 2 (47) to create the protocol. As outlined in the pre-published protocol (https://syrf.org.uk/protocols, 26 March 2020), we extracted information about the strain, sex, age, type of surgical intervention, type of anesthesia and analgesia, time of day, materials used for nest building and burrowing, and video-based analysis of grimace scales vs. direct observation, among other variables. In addition, we obtained information about the respective study quality by application of the SYRCLE risk of bias tool (48), which has been based on the Cochrane risk of bias (49) tool and comprises ten items related to six types of bias to assess the study quality. Reporting was conducted according to the Preferred Reporting Items for Systematic Reviews and Meta-Analyses (PRISMA) guidelines (50). The PRISMA checklist is provided in Supplementary Methods 2. The research question was defined as follows: *What is the current evidence base for using the grimace scale, burrowing, and nest building for the assessment of post-surgical pain in mice and rats?*

### Search string and study selection

A comprehensive search string was developed for PubMed using Entry Terms, keywords, and medical subject headings (MeSH). During search development, “not searches” were performed in which the term being tested and other terms were linked with “not” to evaluate the appropriateness of the term. We combined a search for titles, abstracts, and author-defined keywords with a search for the thesaurus terms. The search comprised relevant synonyms and alternative spellings for the four following components: “tests” (grimace scale, burrowing, nest building), “surgery,” “pain,” and “rodents”.

The following platforms were searched for relevant articles on 16 March 2020: PubMed, Embase *via* Ovid, and Web of Science. The following databases were searched *via* the Web of Science platform: Science Citation Index Expanded, Social Sciences Citation Index, Arts & Humanities Citation Index, Conference Proceedings Citation Index—Science, Conference Proceedings Citation Index—Social Science & Humanities, and Emerging Sources Citation Index. The search string was developed for PubMed and translated to Embase and Web of Science. The four search components were combined within the databases with the Boolean operator “AND.” The final search strings are provided in the protocol and in Supplementary Methods 3.

All search results were transferred to EndNote reference management software (Endnote™ X9). Since, to our knowledge, studies that assess the pain parameters of interest related to post-surgical pain were not published before 2005, and only references from 2005 through 2020 were analyzed. Studies published before 2005 and duplicates were manually removed.

Based on a review by Van der Mierden et al. (51), the web application RAYYAN (52) was selected for the two separate screening phases: title and abstract screening followed by full-text screening. Screeners were trained with the SYRCLE's e-learning tool for preclinical systematic reviews and with a prescreened training set of 50 abstracts. Predefined inclusion and exclusion criteria are presented in Table 1.

**Table 1:** Exclusion criteria defined in the pre-published protocol.

**Prioritized exclusion criteria per selection phase:**
Title-abstract screening
1. No English language 2. No rats and/or mice 3. No surgery, defined as skin incision (including biopsy) under general anesthesia 4. Review article
Full-text screening
1. No English language 2.No rats and/or mice 3. No surgery, defined as skin incision (including biopsy) under general anesthesia 4. No burrowing and/or nest building and/or grimace scale 5. Article without original data 6. Article not retrievable 7. Paper is not a full peer-reviewed journal article

Screening of titles and abstracts was performed by two independent reviewers (KA and VB). In the title and abstract screening, primary studies in English with mice and/or rats describing surgery were included. Surgery was defined as a procedure involving skin incision (including biopsy) under general anesthesia. Discrepancies were resolved by discussion with a third person (IK). In case of remaining doubt about the decision, in this phase, the study was always included.

Two independent reviewers screened for relevant studies during the full-text phase; KA screened the entire set, whereas the set was divided among four independent people as the second reviewer (HS, HK, MB, and CH). In addition to the criteria mentioned above, only full peer-reviewed studies that describe grimace scale, burrowing, and/or nest building as pain parameters were included. Discrepancies were resolved by discussion with a third person (HP). Reference list screening of included studies was conducted by two independent reviewers (KA and HK) to find relevant studies that were not retrieved from the literature databases. Of the references whose title included a surgical intervention and/or the parameters of interest including synonyms, the full text was retrieved and checked for the previously described inclusion criteria. Only full peer-reviewed studies in English describing grimace scale, burrowing, and/or nest building in the post-surgical phase in mice and/or rats were included (refer to Table 1).

The originally described and validated grimace scale for mice (33) comprises five AUs: orbital tightening, nose bulge, cheek bulge, ear position, and whisker change. For rats, Sotocinal et al. (34) initially described and validated four AUs comprising orbital tightening, nose/cheek flattening, ear changes, and whisker change. The term “grimace scale” is used rather liberally in the current literature, and we identified studies either assessing only the eyes or assessing the AUs within a composite behavioral scale. We thus defined the inclusion criterium for “grimace scale” more precisely; at least two AUs had to be scored and that at least one of the following keywords (grimace scale, facial expression, and pain face) had to be mentioned in the text.

### Data extraction

Our unit of analysis was a group of similar animals following the same protocol; if a paper described multiple relevant strains and/ or procedures, data were extracted per group of animals.

Bibliographic details (e.g., first author, year of publication), study design characteristics (e.g., housing, groups), animal model characteristics (e.g., strain, sex), intervention characteristics (e.g., type of surgery, type of analgesia), and outcome measures (e.g., significant alterations of grimace, burrowing or nest building parameters) were extracted for each relevant experimental group/outcome parameter of interest and recorded in an Excel spreadsheet.

For the type of intervention, the following categories were distinguished: biopsy, craniotomy, laparotomy, laparoscopy, (hemi)laminectomy, meniscectomy, neurosurgery, plantar paw incision, subcutaneous implantation, thoracotomy, vascular surgery, and vasectomy. All included procedures grouped under “neurosurgery” were peripheral nervous system interventions.

A subset of 10% of the extracted data, selected by simple randomization with R version 3.6.3. *via* RStudio version 1.2.1335 (53) was quality checked by a second reviewer (K.Sc.).

### Quality assessment—risk of bias

A quality assessment of the studies was performed using SYRCLE's risk of bias (RoB) tool (48). The RoB tool comprises ten items to assess the quality of the included references, which are related to six types of bias: selection bias, performance bias, detection bias, attrition bias, reporting bias, and others. We added the following item: it was assessed whether a power analysis or sample size calculation was reported. Based on signaling questions (refer to Table 2), each item was assessed with the outcome recorded as “YES” (indicates a low risk of bias), “NO” (indicates a high risk of bias), or “UNCLEAR” (indicates an unclear risk of bias). In one of the studies, only one experimental group was used; in this study, selected RoB items were rated “not applicable (NA).” For question Q1 “allocation sequence,” the method of randomization (e.g., randomizer.org, R) had to be defined to result in a low risk of bias evaluation, i.e., reporting “randomly” was insufficient. For question Q10 “other sources of bias,” we focused on the performance of the tests in terms of light and dark phases, the presence of industrial funding, and other suboptimal methods (e.g., picking the best photograph for pain assessment). One reviewer (K.A.) performed the quality assessment of all studies. A random subset of 10% of the extracted data (random selection as for data extraction) was quality checked by a second reviewer (K.Sc.).

**Table 2:** SYRCLE's risk of bias tool signaling questions (48).

**Number**	**Signaling question**
Q1	Was the allocation sequence adequately generated and applied?
Q2	Were the groups similar at baseline?
Q3	Was the allocation adequately concealed?
Q4	Were the animals randomly housed during the experiment?
Q5	Were the caregivers and/or investigators blinded during the experiment?
Q6	Were animals selected at random for outcome assessment?
Q7	Was the outcome assessor blinded?
Q8	Were incomplete outcome data adequately addressed?
Q9	Are reports of the study free of selective outcome reporting?
Q10	Was the study apparently free of other bias?
Q11	Was a power analysis or sample size calculation reported?

Question 11 (Q11) was added.

### Data analysis

Extracted data were separately tabulated for each outcome measure (grimace scale, burrowing, and nest building) in Excel and Word. Thus, a paper describing multiple outcomes is listed in more than one table.

For the evaluation of analgesia and anesthesia, each paper was included once, except for one study that used both mice and rats, which were included as separate studies.

Excel's Pivot tables were used to analyze and plot the data.

Deviating from our protocol, we decided not to perform any meta-analyses in this review, since the heterogeneity in experimental design and outcome parameters between the included studies was considered to be too high.

## Results

### Identification of publications reporting an analysis of the parameters of interest in the post-surgical phase

**Figure 1:**
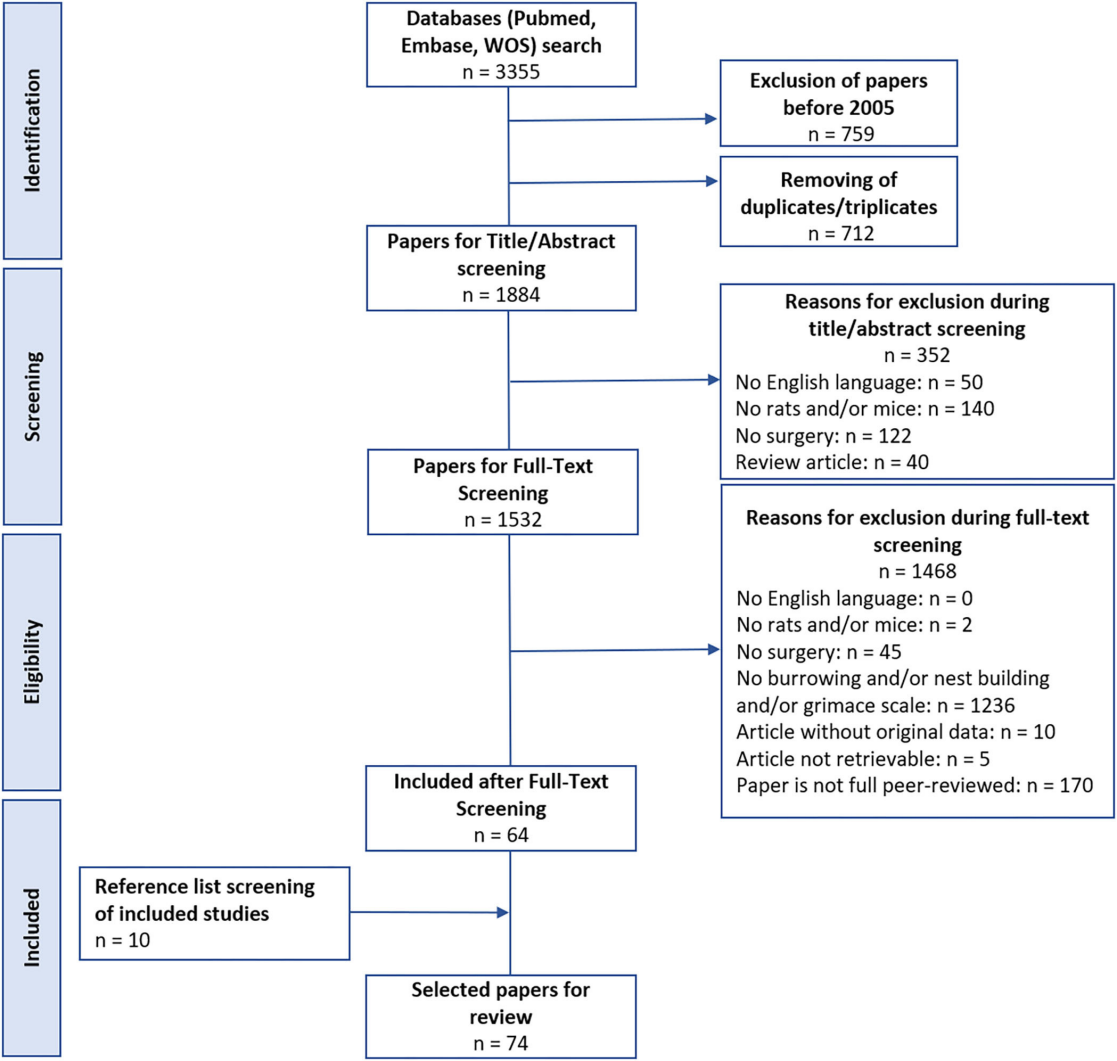
Study selection.

Our searches of the databases retrieved a total of 3,355 papers. Exclusion of studies before 2005 (k = 759) and removing duplicates and triplicates (k = 712) yielded 1,884 papers for screening. In total, 1,532 papers were included after the title and abstract screening, and 64 papers were included after the full-text screening. The reference list screening of included papers revealed 10 additional papers. In total, 74 papers were thus included in the review. A summary of the study flow can be found in Figure 1.

We identified the following number of papers assessing the pain parameters of interest: 18 papers assessing the Mouse Grimace Scale (MGS), 29 papers assessing the Rat Grimace Scale (RGS), 20 papers assessing nest building in mice, one paper assessing nest building in rats, 10 papers assessing burrowing in mice, and seven papers assessing burrowing in rats.

Overall, we included 38 papers using mice as the animal model, 35 papers using rats, and one paper using both rats and mice.

The 38 papers about mice describe 106 study groups, and the 35 papers about rats describe 94 study groups.

Most papers (65/74) evaluated only one pain-associated outcome measure, whereas one paper assessed both grimace scale and nest building, two papers assessed both grimace scale and burrowing, five papers assessed both nest building and burrowing, and only one paper assessed all three pain parameters.

A list of all included papers and evaluated pain parameters is presented in Supplementary Table 1.

### Post-surgical application of mouse and rat grimace scales

**Figure 2:**
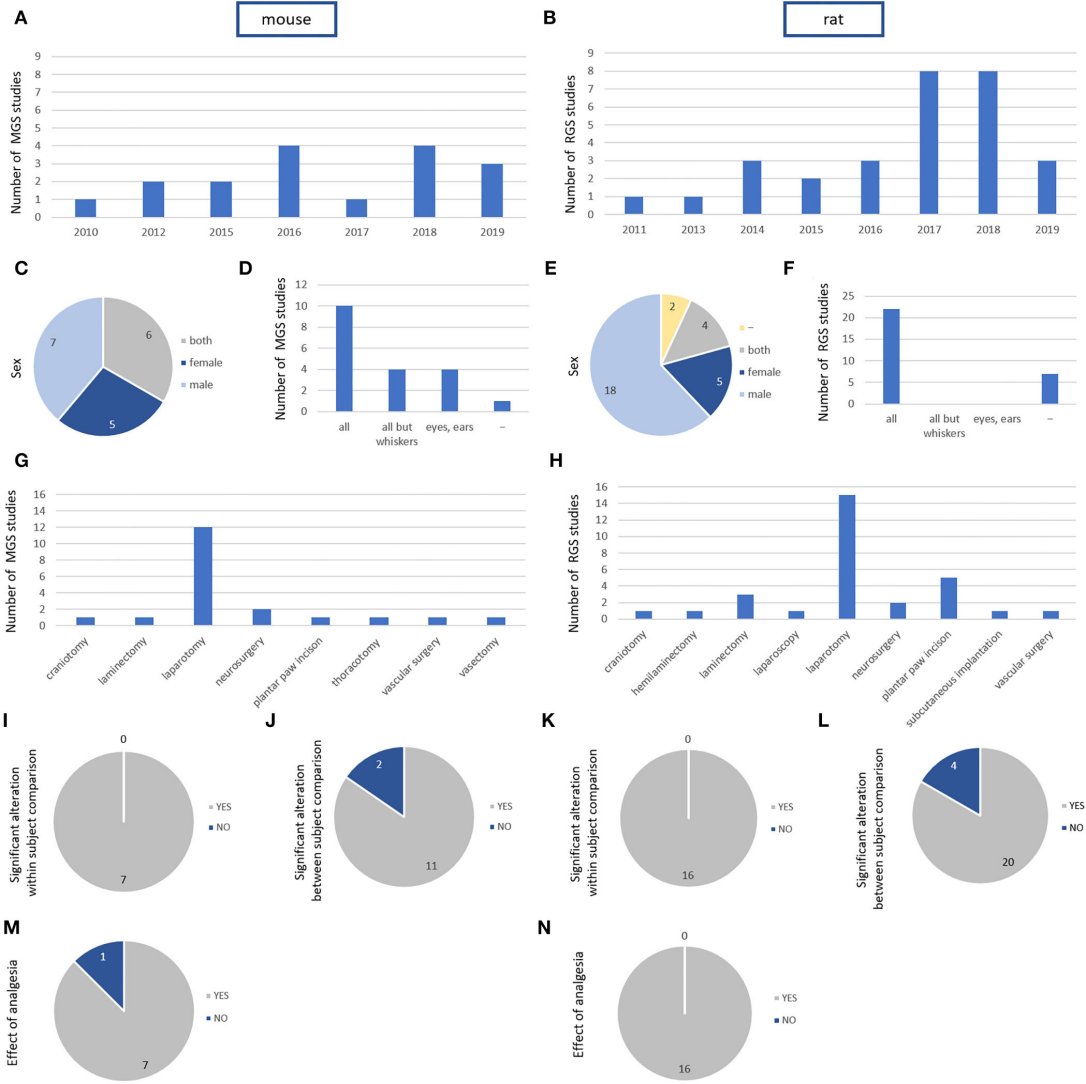
Study characteristics, animal characteristics, and intervention characteristics for Mouse Grimace Scale (k = 18 studies) and Rat Grimace Scale (k = 29 studies). **(A,B)** Number of published studies using grimace scale for the assessment of post-surgical pain in mice **(A)** and rats **(B)** during the last decade. X-axis represents years. **(C,E)** Number of grimace scale studies in mice **(C)** and rats **(E)** using females, males, and both sexes. **(D,F)** Scored action units (AUs) in mice **(D)** and rats **(F)**. All but whisker: all AUs were scored except of whisker change. Eyes, ears: narrowing of the eyes and ears changes were evaluated. **(G,H)** Type of intervention in mice **(G)** and rats **(H)**. **(I,K)** Studies describing within-subject comparison in mice **(I)** and rats **(K)**. The within-subject comparison revealed an influence of the surgical intervention in all mouse and all rat studies. **(J,L)** Studies describing between-subject comparison in mice **(J)** and rats **(L)**. **(M,N)** Studies describing an analysis of the impact of an analgetic drug on MGS **(M)** or RGS **(N)** in comparison with a control group. The analysis revealed an influence in all rat studies.

Screening identified 18 mouse (25, 33, 54–69) and 29 rat (24, 34, 55, 70–95) studies published between 2010 and 2020 that explored grimace scales in the post-surgical phase. The countries of origin of the first author comprise the United Kingdom and different European and North American countries for studies with an analysis of MGS. For rat studies, the respective list of countries includes Asia, Europe, North, and South America (Table 3). The first post-surgical grimace scale study in mice (MGS) was published in 2010; the first was in rats (RGS) in 2011 (Figures 2A,B).

**Table 3:** Study characteristics and animal characteristics for Mouse Grimace Scale (k = 18 studies) and Rat Grimace Scale (k = 29 studies).

**Study ID**	**Year of publication**	**Country of origin first author**	**Strain**	**Breeder**	**Sex**	**Age arrival [weeks]**	**Body weight on arrival [g]**	**Body weight on evaluation [g]**
**Mice**								
Akintola et al. (55)	2017	USA	C57BL/6J	Jackson laboratories	Male	10–12	–	–
Cho et al. (54)	2019	Canada	Crl:CD1(ICR)	In-House, Charles River	Both	6–8	–	–
Cho et al. (54)	2019	Canada	C57BL/6N	Charles River	Both	6–8	–	–
Dwivedi et al. (69)^+^	2016	Canada	C57BL/6J	Jackson laboratories	Both	10–12	–	–
Evangelista-Vaz et al. (56)	2018	Switzerland	C57BL/6J	Charles River	Female	6–8	18–22	–
Faller et al. (57)	2015	UK	C57BL/6J	Harlan UK	Female	–	–	–
Gallo et al. (58)	2019	USA	Crl:CD1(ICR)	Charles River	Male	8–9	–	–
Hsi et al. (60)	2020	USA	–	–	Both	7–9	25.5–44.7	–
Jirkof et al. (59)	2015	Switzerland	C57BL/6J	Charles River	Female	6–8	–	–
Jirkof et al. (61)	2018	Switzerland	C57BL/6J	Charles River	Female	6–8	–	–
Langford et al. (33)	2010	Canada	Crl:CD1(ICR)	In-House breeding	Both	6–18	–	–
Leach et al. (25)	2012	UK	Crl:CD1(ICR)	Charles River	Male	–	30–40	–
Mai et al. (63)	2018	Canada	C57BL/6J	Charles River	Male	8–12	20–25	–
Matsumiya et al. (64)	2012	Canada	Crl:CD1(ICR)	Charles River	Both	6–8	–	–
Miller et al. (62)	2016	UK	CBA	Charles River	Male	–	25.6–28.7	–
Redaelli et al. (67)	2019	Italy	C57BL/6N	Charles River	Male	8–9	25	–
Roughan et al. (65)	2016	UK	BALB/C	Charles River	Male	–	25–30	–
Sauer et al. (68)^+^	2016	Switzerland	C57BL/6J	Charles River	Female	4–5	–	–
Tuttle et al. (66)	2018	USA	Crl:CD1(ICR)	Charles River	Both	6–12	–	–
**Rats**								
Akintola et al. (55)	2017	USA	SD	Envigo	Male	10–13	–	–
Chaves et al. (71)	2018	Brazil	Wistar	Animal Colony of the Instituto Evandro Chagas	Male	13–17	250–350	–
Chi et al. (72)	2013	Japan	Wistar	–	Male	52–56	550–640	–
Clemensen et al. (73)	2018	Denmark	SD	Taconic	Male	8	296–302	–
De Rantere et al. (74)	2018	Canada	Wistar	Charles River	Male	8	–	–
Fujita et al. (75)	2018	Japan	SD	SLC Ltd	Male	–	–	246–274
Gao et al. (76)	2017	China	Wistar	Animal house of Beijing Shijitan Hospital	–	9–11	275–325	–
Guo and Hu (77)	2017	China	Wistar	–	Male	104–108	550–640	–
Harikrishnan et al. (78)	2019	India	Wistar	Charles River	Female	9–12	240–280	–
Jeger et al. (94)^+^	2017	Switzerland	Wistar	–	Male	–	340–492	–
Kawano et al. (80)	2014	Japan	Wistar	–	Male	104–108	–	–
Kawano et al. (79)	2017	Japan	SD	–	Male	–	–	–
Kawano et al. (93)^+^	2018	Japan	Wistar	–	Male	9–17	–	–
Kawano et al. (93)^+^			Wistar	–	Male	83–96	–	–
Klune et al. (24)	2019	USA	Wistar and SD	Charles River	Female	6	150–350	–
Korat et al. (81)	2017	India	–	In-House	Both	–	267–310	–
Korat and Kapupara (70)	2018	India	–	In-House	Both	–	265–315	–
Koyama et al. (82)	2019	Japan	Wistar	–	Male	9–17	–	–
Koyama et al. (82)	2019	Japan	Wistar	–	Male	80–104	–	–
Locatelli et al. (95)^+^	2018	Japan	Wistar	Alfresa Shinohara Chemicals Corporation	Male	83–96	–	–
Nunamaker et al. (83)	2018	USA	SD	Envigo	Female	–	199.6–215	–
Oliver et al. (84)	2014	Canada	SD	in-house	Female	–	284–420	–
Philips et al. (85)	2016	USA	SD	Harlan	Male	–	275–349	–
Prefontaine et al. (91)	2014	Canada	SD	Charles River	Male	–	275–325	–
Saine et al. (86)	2016	Canada	SD	Charles River	Male	–	300–380	–
Schneider et al. (92)^+^	2017	USA	SD	Charles River	Male	–	275–300	–
Sotocinal et al. (34)	2011	Canada	Wistar	Charles River	Both	6–8	200–250	–
Thomas et al. (87)	2016	UK	Wistar	Charles River	Female	58–64	270	–
Waite et al. (88)	2015	USA	Wistar	Charles River	Both	–	250–300	–
Yamanaka et al. (89)	2017	Japan	Wistar	–	Male	5–6	135–180	–
Yousef et al. (90)	2015	Italy	SD	–	–	–	225–250	–

–not reported. Alphabetical characters in the Study ID indicate animal groups within individual studies [first column of the tables; e.g., Cho et al. (54), Koyama et al. (93)]. ^+^References were identified during screening of reference lists.

For both scores, an increase in the application was observed at the end of the decade (Figures 2A,B). Since the search in the databases was conducted in early 2020, the year 2020 was not included in the graphs.

While there was only a slight imbalance in the use of male and female animals in mouse studies, the majority of rat studies were conducted in male animals, totaling 62% (18/29 studies; Table 3, Figures 2C,E).

Information about the mouse and rat strains used could be extracted from the majority of papers (Table 3). The analysis revealed a predominance of studies in C57BL/6J mice (eight studies in total). The next most frequently used mouse strain was Crl:CD1(ICR). For further mouse strains reported in the identified publications, the number of studies per strain was one or two. All rat studies, except two where the strain was not reported, were conducted in Wistar rats (k = 16) and/or Sprague Dawley rats (k = 12).

Assuming a frequently applied habituation phase of 1–2 weeks following arrival, age on arrival indicates that the majority of studies (13/18) in mice were completed in young adult mice (Table 3). The exact body weight at the time of the surgical intervention or MGS assessment was not reported in all studies. In only one study, younger mice (<6 w; age 4–5 weeks on arrival) were ordered from the commercial breeder (68). In this study, animals were 7–8 weeks old during testing.

Another study reported an age of 6–18 weeks on arrival, indicating the use of adult mice in one of the subprojects (33). However, in this case, it was impossible to conclude the exact age at which the intervention was conducted.

For rats, a broader age range was indicated from the information about age on arrival ranging from 5 to 108 weeks. This indicated that studies were performed in young adult, adult, and aged rats (Table 3).

Whereas the majority of studies in rats considered all AUs, several studies in mice did not apply the whisker score (4/18 studies) or instead focused on orbital tightening and ear position (4/18 studies). The remaining studies in mice included all AUs in their analysis (10/18 studies). In Cho et al. (54), all AUs were examined in CD-1 mice, whereas in BL6 mice, all AUs were examined except whiskers due to poor visibility. A video- or image-based analysis was reported in 61% of the mouse and 72% of the rat studies (Table 4, Figures 2D,F).

**Table 4:** Outcome characteristics for Mouse Grimace Scale (k = 18 studies) and Rat Grimace Scale (k = 29 studies).

**Study ID**	**Scored individual action units**	**Range of scores**	**Video/Image based evaluation**	**Time of day** **during evaluation**	**Baseline**	**Significant alteration of grimace scale parameters**		**Evaluated parameter/Comments**
						**Within-subject comparison**	**Between-subject comparison**	
**Mice**								
Akintola et al. (55)	All	0–2	Yes	–	Yes	Yes	–	Mean score
Cho et al. (54)	All	–	Yes	–	Yes	–	Yes	Mean difference score
Cho et al. (54)	All but whiskers	–	Yes	–	Yes	–	Yes	Mean difference score
Dwivedi et al. (69)^+^	–	0–3	–	–	–	–	Yes	Mean score
Evangelista-Vaz et al. (56)	Orbital tightening, ear position	0–2	No	–	–	–	Yes	Composite behavioral score
Faller et al. (57)	All	0–2	Yes	–	–	–	Yes	Mean score
Gallo et al. (58)	All	0–2	Yes	07 a.m. to 10 a.m.	Yes	Yes	–	
Hsi et al. (60)	All	0–2	–	–	–	–	No	Mean score
Jirkof et al. (59)	Orbital tightening, ear position	0–2	–	–	–	–	Yes	Composite behavioral score
Jirkof et al. (61)	Orbital tightening, ear position	–	–	–	–	–	Yes	Composite behavioral score
Langford et al. (33)	All	0–2	Yes	–	Yes	Yes	–	Mean difference score
Leach et al. (25)	All but whiskers	0–2	Yes	–	Yes	Yes	Yes	Mean score
Mai et al. (63)	All	0–3	–	–	–	–	Yes	Mean score
Matsumiya et al. (64)	All	0–2	Yes	–	Yes	–	Yes	Mean difference score
Miller et al. (62)	All but whiskers	–	Yes	–	Yes	Yes	–	Mean score
Redaelli et al. (67)	All	0–2	Yes	–	Yes	Yes	Yes	Mean score
Roughan et al. (65)	All but whiskers	0–2	Yes	–	Yes	Yes	No	Mean score
Sauer et al. (68)^+^	Orbital tightening, ear position	0–2	–	–	–	–	–	Composite behavioral score
Tuttle et al. (66)	All	0–2	Yes	–	Yes	–	Yes	Mean score, Mean difference score
**Rats**								
Akintola et al. (55)	All	0–2	Yes	–	Yes	Yes	No	Mean score
Chaves et al. (71)	All	0–2	Yes	–	–	Yes	Yes	Mean score
Chi et al. (72)	All	0–2	Yes	–	Yes	Yes	–	Mean score
Clemensen et al. (73)	All	0–2	No	–	Yes	–	Yes	Mean score
De Rantere et al. (74)	All	0–2	Yes	–	Yes	Yes	Yes	Mean score
Fujita et al. (75)	All	0–8	–	–	–	–	No	
Gao et al. (76)	–	0–2	Yes	–	Yes	Yes	Yes	Mean score
Guo and Hu (77)	All	0–2	Yes	–	Yes	–	Yes	Mean score
Harikrishnan et al. (78)	All	–	Yes	09 a.m. to 3 p.m.	Yes	–	Yes	Mean difference score
Jeger et al. (94)^+^	All	0–2	–	–	–	–	Yes	Mean score
Kawano et al. (80)	All	0–2	–	–	Yes	Yes	–	Mean score
Kawano et al. (79)	All	0–2	Yes	–	Yes	–	Yes	Mean score
Kawano et al. (93)^+^	–	0–2	–	–	–	–	No	Mean score
Klune et al. (24)	All	0–2	Yes	–	Yes	Yes	Yes	Mean score
Korat et al. (81)	–	0–4	Yes	–	Yes	Yes	Yes	Mean score
Korat and Kapupara (70)	–	0–3	Yes	–	Yes	Yes	Yes	Total score
Koyama et al. (82)	–	0–2	–	–	Yes	Yes	–	Mean score
Locatelli et al. (95)^+^	–	–	–	–	–	–	No	Mean score
Nunamaker et al. (83)	All	0–2	Yes	–	Yes	–	Yes	Mean score
Oliver et al. (84)	All	0–2	Yes	–	Yes	Yes	–	Mean score
Philips et al. (85)	All	0–2	Yes	–	Yes	Yes	Yes	Mean score
Prefontaine et al. (91)	All	0–1	Yes	–	–	–	Yes	Mean score
Saine et al. (86)	All	0–2	Yes	–	Yes	–	Yes	Mean score
Schneider et al. (92)^+^	All	0–2	Yes	–	Yes	Yes	Yes	Mean score
Sotocinal et al. (34)	All	0–2	Yes	–	Yes	Yes	Yes	Mean score
Thomas et al. (87)	All	0–2	Yes	–	Yes	Yes	Yes	Mean score
Waite et al. (88)	All	–	Yes	–	Yes	–	Yes	Mean difference score
Yamanaka et al. (89)	All	0–2	Yes	–	Yes	Yes	Yes	Mean score
Yousef et al. (90)	All	0–2	–	–	–	–	–	Mean score

–not reported. Alphabetical characters in the Study ID indicate animal groups within individual studies [first column of the tables; e.g., Cho et al. (54)]. ^+^References were identified during screening of reference lists.

Concerning the type of surgical intervention, laparotomy was the most frequently performed procedure, accounting for 67% of mouse and 52% of rat studies identified. Other interventions in mice included craniotomy, neurosurgery, laminectomy, thoracotomy, vascular surgery, plantar paw incision, and vasectomy. In rat studies, the list of further surgical techniques, in addition to the aforementioned, included hemilaminectomy, laparoscopy, and subcutaneous implantation (Figures 2G,H).

Baseline data were collected in 10 of the 18 mouse studies and 22 of the 29 rat studies. A comparison with baseline levels (within-subject design) was described in seven of the mouse studies and 16 of the rat studies. The within-subject comparison revealed an influence in seven of the mouse and 16 of the rat studies (Table 4, Figures 2I,K).

An impact of the surgical intervention on MGS and RGS based on a between-subject design was assessed in 13 mouse and 24 rat studies. The between-group comparison confirmed an effect in 11 and 20 of the mouse and rat studies (Table 4, Figures 2J,L).

An impact of a single analgetic or combination of analgetic drugs on MGS and RGS in comparison with the control group was analyzed in 8 of 18 mouse studies and 16 of 29 rat studies. The analysis revealed an impact in 7 mouse and 16 rat studies (Table 5, Figures 2M,N). The most frequently used drugs included NSAIDs and opioids, most often administered subcutaneously or intraperitoneally.

**Table 5:** Design and intervention characteristics for grimace scale, nest building, and burrowing in mice (k = 39 studies) and rats (k = 36 studies).

**Study ID**	**Groups**	**Housing**	***n*/Home cage**	**Type of surgery**	**Type of anesthesia**	**Type of analgesia 1**	**Route**	**Dose (mg/kg)**	**Type of analgesia 2**	**Route**	**Dose (mg/kg)**	Effect of analgesia*
**Grimace scale mice**												
Akintola et al. (55)	CCI-ION	Group	4–6	Neurosurgery	Ketamine and xylazine	–	–	–	–	–	–	–
Cho et al. (54)	Carprofen 10 mg/kg	Single before surgery	1	Craniotomy	Isoflurane	Carprofen	s.c.	10	–	–	–	Yes
Cho et al. (54)	Carprofen 25 mg/kg	Single before surgery	1	Craniotomy	Isoflurane	Carprofen	s.c.	25	–	–	–	Yes
Cho et al. (54)	Meloxicam 2 mg/kg	Single before surgery	1	Craniotomy	Isoflurane	Meloxicam	s.c.	2	–	–	–	Yes
Cho et al. (54)	Meloxicam 5 mg/kg	Single before surgery	1	Craniotomy	Isoflurane	Meloxicam	s.c.	5	–	–	–	Yes
Cho et al. (54)	Buprenorphine 0.1 mg/kg	Single before surgery	1	Craniotomy	Isoflurane	Buprenorphine	s.c.	0.1	–	–	–	Yes
Cho et al. (54)	Carprofen 10 mg/kg	Single before surgery	1	Craniotomy	Isoflurane	Carprofen	Oral (drinking supply)	10	–	–	–	Yes
Cho et al. (54)	Carprofen 25 mg/kg	Single before surgery	1	Craniotomy	Isoflurane	Carprofen	Oral (drinking supply)	25	–	–	–	Yes
Cho et al. (54)	Meloxicam 2 mg/kg	Single before surgery	1	Craniotomy	Isoflurane	Meloxicam	Oral (drinking supply)	2	–	–	–	Yes
Cho et al. (54)	Meloxicam 5mg/kg	Single before surgery	1	Craniotomy	Isoflurane	Meloxicam	Oral (drinking supply)	5	–	–	–	Yes
Cho et al. (54)	Buprenorphine 0.1 mg/kg	Single before surgery	1	Craniotomy	Isoflurane	Buprenorphine	Oral (drinking supply)	0.1	–	–	–	Yes
Dwivedi et al. (69)^+^	Laparotomy + CLP	–	–	Laparotomy	Isoflurane	Buprenorphine	s.c.	0.1	–	–	–	–
Evangelista-Vaz et al. (56)	Surgery + anesthesia + tramadol injection + drinking supply	Group	4–8	Laparotomy	Sevoflurane	Tramadol	s.c.	25	Tramadol	Oral (drinking supply)	25	No
Faller et al. (57)	Myocardial infarction	Group	2–5	Thoracotomy	Isoflurane	Buprenorphine	s.c.	0.024	–	–	–	–
Gallo et al. (58)	Nest material + surgery + analgesia	Single before surgery	1	Vascular surgery	Ketamine and xylazine	Buprenorphine	s.c.	0.05	–	–	–	–
Hsi et al. (60)	Surgery + dextrose (dose group)	Single before surgery	1	Laparotomy	Isoflurane	Meloxicam	s.c.	2	Buprenorphine	s.c.	0.1	–
Jirkof et al. (59)	OPT3 (surgery + anesthesia + T3)	Group	3–6	Laparotomy	Sevoflurane	Buprenorphine	s.c.	0.1	–	–	–	Yes
Jirkof et al. (59)	OPSB (surgery + anesthesia + SB)	Group	3–6	Laparotomy	Sevoflurane	Buprenorphine SR	s.c.	2.2	–	–	–	Yes
Jirkof et al. (61)	Anesthesia and surgery with T:P in the drinking water	Single before surgery	1	Laparotomy	Sevoflurane	Tramadol	Oral (drinking supply)	–	Paracetamol	Oral (drinking supply)	–	Yes
Langford et al. (33)	Chronic constriction injury (CCI)	Group	2	Neurosurgery	–	–	–	–	–	–	–	–
Langford et al. (33)	Incision model	Group	2	Plantar paw incision	Isoflurane	–	–	–	–	–	–	–
Langford et al. (33)	Laparotomy model	Group	2	Laparotomy	Isoflurane	–	–	–	–	–	–	–
Langford et al. (33)	Spared nerve injury (SNI)	Group	2	Neurosurgery	–	–	–	–	–	–	–	–
Leach et al. (25)	Surgery + meloxicam	Single before surgery	1	Vasectomy	Isoflurane	Meloxicam	s.c.	20	Buprenorphine	s.c.	0.05	Yes
Leach et al. (25)	Surgery + bupivacaine	Single before surgery	1	Vasectomy	Isoflurane	Bupivacaine	Wound infiltration	5	Buprenorphine	s.c.	0.05	Yes
Mai et al. (63)	Severe CLP	Group	3	Laparotomy	Isoflurane	Buprenorphine	s.c.	0.1	–	–	–	–
Matsumiya et al. (64)	Laparotomy + buprenorphine 0.001	Single from surgery on	1	Laparotomy	Isoflurane	Buprenorphine	s.c.	0.001	–	–	–	Yes
Matsumiya et al. (64)	Laparotomy + buprenorphine 0.01	Single from surgery on	1	Laparotomy	Isoflurane	Buprenorphine	s.c.	0.01	–	–	–	Yes
Matsumiya et al. (64)	Laparotomy + buprenorphine 0.05	Single from surgery on	1	Laparotomy	Isoflurane	Buprenorphine	s.c.	0.05	–	–	–	Yes
Matsumiya et al. (64)	Laparotomy + buprenorphine 0.1	Single from surgery on	1	Laparotomy	Isoflurane	Buprenorphine	s.c.	0.1	–	–	–	Yes
Matsumiya et al. (64)	Laparotomy + carprofen 5	Single from surgery on	1	Laparotomy	Isoflurane	Carprofen	s.c.	5	–	–	–	Yes
Matsumiya et al. (64)	Laparotomy + carprofen 10	Single from surgery on	1	Laparotomy	Isoflurane	Carprofen	s.c.	10	–	–	–	Yes
Matsumiya et al. (64)	Laparotomy + carprofen 15	Single from surgery on	1	Laparotomy	Isoflurane	Carprofen	s.c.	15	–	–	–	Yes
Matsumiya et al. (64)	Laparotomy + carprofen 20	Single from surgery on	1	Laparotomy	Isoflurane	Carprofen	s.c.	20	–	–	–	Yes
Matsumiya et al. (64)	Laparotomy + carprofen 25	Single from surgery on	1	Laparotomy	Isoflurane	Carprofen	s.c.	25	–	–	–	Yes
Matsumiya et al. (64)	Laparotomy + ketoprofen 1	Single from surgery on	1	Laparotomy	Isoflurane	Ketoprofen	s.c.	1	–	–	–	Yes
Matsumiya et al. (64)	Laparotomy + ketoprofen 5	Single from surgery on	1	Laparotomy	Isoflurane	Ketoprofen	s.c.	5	–	–	–	Yes
Matsumiya et al. (64)	Laparotomy + ketoprofen 10	Single from surgery on	1	Laparotomy	Isoflurane	Ketoprofen	s.c.	10	–	–	–	Yes
Matsumiya et al. (64)	Laparotomy + ketoprofen 15	Single from surgery on	1	Laparotomy	Isoflurane	Ketoprofen	s.c.	15	–	–	–	Yes
Matsumiya et al. (64)	Laparotomy + ketoprofen 20	Single from surgery on	1	Laparotomy	Isoflurane	Ketoprofen	s.c.	20	–	–	–	Yes
Matsumiya et al. (64)	Laparotomy + acetaminophen 100	Single from surgery on	1	Laparotomy	Isoflurane	Acetaminophen	s.c.	100	–	–	–	Yes
Matsumiya et al. (64)	Laparotomy + acetaminophen 300	Single from surgery on	1	Laparotomy	Isoflurane	Acetaminophen	s.c.	300	–	–	–	Yes
Matsumiya et al. (64)	Laparotomy + acetaminophen 450	Single from surgery on	1	Laparotomy	Isoflurane	Acetaminophen	s.c.	450	–	–	–	Yes
Miller et al. (62)	Study group	Group	4	Laparotomy	Isoflurane	Buprenorphine	s.c.	0.05	Meloxicam	s.c.	5	–
Redaelli et al. (67)	Surgery + buprenorphine (step 2; control for carprofen)	Group	2	Laminectomy	Isoflurane	Buprenorphine	s.c.	0.15	–	–	–	Yes
Redaelli et al. (67)	Surgery + buprenorphine + carprofen (step2)	Group	2	Laminectomy	Isoflurane	Buprenorphine	s.c.	0.15	Carprofen	s.c.	5	Yes
Roughan et al. (65)	Laparotomy + meloxicam 1 mg/kg	Group	5	Laparotomy	Isoflurane	Meloxicam	s.c.	1	–	–	–	–
Roughan et al. (65)	Laparotomy + meloxicam 5 mg/kg	Group	5	Laparotomy	Isoflurane	Meloxicam	s.c.	5	–	–	–	–
Roughan et al. (65)	Laparotomy + meloxicam 20 mg/kg	Group	5	Laparotomy	Isoflurane	Meloxicam	s.c.	20	–	–	–	–
Sauer et al. (68)^+^	Surgery + buprenorphine *via* 3 injections and *via* drinking water	Group	4–8	Laparotomy	Sevoflurane	Buprenorphine	s.c.	0.1	Buprenorphine	Oral (drinking supply)	–	–
Tuttle et al. (66)	Laparotomy + carprofen	Group	5	Laparotomy	Isoflurane	Carprofen	s.c.	50	–	–	–	Yes
**Grimace scale rats**												
Akintola et al. (55)	CCI-ION (RGS post 10 days)	Group	2	Neurosurgery	Ketamine and xylazine	Fentanyl	s.c.	0.025	–	–	–	–
Chaves et al. (71)	Laminectomy	Single from surgery on	–	Laminectomy	Ketamine and xylazine	Fentanyl	i.p.	0.03	–	–	–	–
Chaves et al. (71)	Laminectomy + tramadol	Single from surgery on	–	Laminectomy	Ketamine and xylazine	Fentanyl	i.p.	0.03	Tramadol	s.c.	4	–
Chi et al. (72)	Isoflurane + laparotomy + ropivacaine	–	–	Laparotomy	Isoflurane	Ropivacaine	Wound infiltration	–	–	–	–	Yes
Chi et al. (72)	Isoflurane + laparotomy + morphine	–	–	Laparotomy	Isoflurane	Morphine	s.c.	0.8	–	–	–	Yes
Clemensen et al. (73)	Hind-Paw incision + low dose fentanyl	Single before surgery	1	Plantar paw incision	Isoflurane	Fentanyl	Transdermal	0.1	–	–	–	Yes
Clemensen et al. (73)	Hind-Paw incision + middle dose fentanyl	Single before surgery	1	Plantar paw incision	Isoflurane	Fentanyl	Transdermal	0.33	–	–	–	Yes
Clemensen et al. (73)	Hind-Paw incision + high dose fentanyl	Single before surgery	1	Plantar paw incision	Isoflurane	Fentanyl	Transdermal	1	–	–	–	Yes
De Rantere et al. (74)	Plantar incision	Group	2	Plantar paw incision	Isoflurane	–	–	–	–	–	–	–
Fujita et al. (75)	Allopregnanolone	–	–	Plantar paw incision	Isoflurane	–	–	–	–	–	–	–
Gao et al. (76)	Treatment	Group	2	Laparotomy	Isoflurane	Solution containing Levobupivacaine, Dexibuprofen, Norepinephrine	Wound infiltration	–	–	–	–	Yes
Gao et al. (76)	Positive control	Group	2	Laparotomy	Isoflurane	Solution containing Levobupivacaine, Dexibuprofen, Norepinephrine	Systematically	–	–	–	–	Yes
Guo and Hu (77)	Anesthesia + laparotomy + Thalidomide 5 mg/kg	–	–	Laparotomy	Isoflurane	Thalidomide	i.p.	5	–	–	–	Yes
Guo and Hu (77)	Anesthesia + laparotomy + thalidomide 20 mg/kg	–	–	Laparotomy	Isoflurane	Thalidomide	i.p.	20	–	–	–	Yes
Guo and Hu (77)	Anesthesia + laparotomy + thalidomide 50 mg/kg	–	–	Laparotomy	Isoflurane	Thalidomide	i.p.	50	–	–	–	Yes
Harikrishnan et al. (78)	Laminectomy without SCI dental burr assisted (DBA-LAM)	Single before surgery	1	Laminectomy	Isoflurane	Buprenorphine	s.c.	0.05	Meloxicam	s.c.	1	–
Jeger et al. (94)^+^	Long term + surgery + sham + nalbuphine	Single from surgery on	3–4	Vascular surgery	Isoflurane	Nalbuphine	s.c.	2	Nalbuphine	i.v.	1	Yes
Kawano et al. (80)	Anesthesia with laparotomy and ketoprofen (IL + ketoprofen)	–	–	Laparotomy	Isoflurane	Ketoprofen	s.c.	40	–	–	–	Yes
Kawano et al. (80)	Anesthesia with laparotomy and morphine (IL + morphine)	–	–	Laparotomy	Isoflurane	Morphine	s.c.	0.8	–	–	–	Yes
Kawano et al. (79)	Surgery + control + ketoprofen 5	Single before surgery	–	Plantar paw incision	Isoflurane	Ketoprofen	i.p.	5	–	–	–	Yes
Kawano et al. (79)	Surgery + control + ketoprofen 10	Single before surgery	–	Plantar paw incision	Isoflurane	Ketoprofen	i.p.	10	–	–	–	Yes
Kawano et al. (79)	Surgery + control + ketoprofen 15	Single before surgery	–	Plantar paw incision	Isoflurane	Ketoprofen	i.p.	15	–	–	–	Yes
Kawano et al. (79)	Surgery + control + ketoprofen 30	Single before surgery	–	Plantar paw incision	Isoflurane	Ketoprofen	i.p.	30	–	–	–	Yes
Kawano et al. (79)	Surgery + control + morphine 0.1	Single before surgery	–	Plantar paw incision	Isoflurane	Morphine	i.p.	0.1	–	–	–	Yes
Kawano et al. (79)	Surgery + control + morphine 0.5	Single before surgery	–	Plantar paw incision	Isoflurane	Morphine	i.p.	0.5	–	–	–	Yes
Kawano et al. (79)	Surgery + control + morphine 1.0	Single before surgery	–	Plantar paw incision	Isoflurane	Morphine	i.p.	1	–	–	–	Yes
Kawano et al. (79)	Surgery + control + morphine 1.5	Single before surgery	–	Plantar paw incision	Isoflurane	Morphine	i.p.	1.5	–	–	–	Yes
Kawano et al. (79)	Surgery + control + ropivacaine	Single before surgery	–	Plantar paw incision	Isoflurane	Ropivacaine	Wound infiltration	–	–	–	–	Yes
Kawano et al. (93)^+^	Isoflurane with laparotomy	Group	–	Laparotomy	Isoflurane	Ropivacaine	–	–	–	–	–	–
Klune et al. (24)	Laparotomy + meloxicam	Group	2	Laparotomy	Isoflurane	Meloxicam	s.c.	2	–	–	–	Yes
Klune et al. (24)	Laparotomy + buprenorphine	Group	2	Laparotomy	Isoflurane	Buprenorphine	s.c.	0.05	–	–	–	Yes
Korat et al. (81)	Treatment	Group	2	Laparotomy	Isoflurane	Solution containing levobupivacaine, ibuprofen and epinephrine	Wound infiltration	–	–	–	–	Yes
Korat and Kapupara (70)	Experimental group	Group	2	Laparotomy	Isoflurane	Solution containing levobupivacaine, ibuprofen and epinephrine	Wound infiltration	–	–	–	–	Yes
Korat and Kapupara (70)	Experimental group	Group	2	Laparotomy	Isoflurane	Solution containing levobupivacaine, ibuprofen and epinephrine	i.p.	–	–	–	–	Yes
Koyama et al. (82)	Anesthesia with surgery + ropivacaine	–	–	Laparotomy	Isoflurane	Ropivacaine	Wound infiltration	–	–	–	–	Yes
Koyama et al. (82)	Anesthesia with surgery + morphine	–	–	Laparotomy	Isoflurane	Morphine	s.c.	0.8	–	–	–	Yes
Locatelli et al. (95)^+^	Surgery + 80 mg/kg e-RESV + sirtinol	–	–	Laparotomy	–	Ropivacaine	–	–	–	–	–	–
Nunamaker et al. (83)	Surgery + meloxicam low dose	Single before surgery	1	Laparotomy	Ketamine	Meloxicam	s.c.	1	–	–	–	Yes
Nunamaker et al. (83)	Surgery + meloxicam high dose	Single before surgery	1	Laparotomy	Ketamine	Meloxicam	s.c.	2	–	–	–	Yes
Nunamaker et al. (83)	Surgery + buprenorphine low dose	Single before surgery	1	Laparotomy	Ketamine	Buprenorphine	s.c.	0.05	–	–	–	Yes
Nunamaker et al. (83)	Surgery + buprenorphine high dose	Single before surgery	1	Laparotomy	Ketamine	Buprenorphine	s.c.	0.1	–	–	–	Yes
Nunamaker et al. (83)	Surgery + SRB	Single before surgery	1	Laparotomy	Ketamine	Buprenorphine SR	s.c.	1.2	–	–	–	Yes
Oliver et al. (84)	Surgery + buprenorphine s.c.	Group	2–3	Subcutaneous implantation	Isoflurane	Lidocaine	s.c.	2	Buprenorphine	s.c.	0.05	–
Oliver et al. (84)	Surgery + buprenorphine oral	Group	2–3	Subcutaneous implantation	Isoflurane	Lidocaine	s.c.	2	Buprenorphine	p.o.	0.05	–
Oliver et al. (84)	Surgery + meloxicam	Group	2–3	Subcutaneous implantation	Isoflurane	Lidocaine	s.c.	2	Meloxicam	s.c.	1	–
Philips et al. (85)	Hemilaminectomy + nerve root compression + meloxicam	Group	2	Hemi-Laminectomy	Isoflurane	Meloxicam	s.c.	2	–	–	–	Yes
Prefontaine et al. (91)	Laparoscopy	Group	2	Laparoscopy	Isoflurane	–	–	–	–	–	–	–
Prefontaine et al. (91)	Laparotomy	Group	2	Laparotomy	Isoflurane	–	–	–	–	–	–	–
Saine et al. (86)	Craniotomy + collagenase i.c. + fentanyl 5	Group	2	Craniotomy	Isoflurane	Fentanyl	s.c.	5	–	–	–	Yes
Saine et al. (86)	Craniotomy + collagenase i.c. + Fentanyl 10	Group	2	Craniotomy	Isoflurane	Fentanyl	s.c.	10	–	–	–	Yes
Saine et al. (86)	Craniotomy + collagenase i.c. + Fentanyl 20	Group	2	Craniotomy	Isoflurane	Fentanyl	s.c.	20	–	–	–	Yes
Schneider et al. (92)^+^	Laminectomy + cervical SCI	Group	2–3	Laminectomy	Isoflurane	Carprofen	s.c.	5	–	–	–	–
Sotocinal et al. (34)	Laparotomy model	Group	2	Laparotomy	Isoflurane	–	–	–	–	–	–	–
Thomas et al. (87)	Laparotomy + Morphine s.c.	Group	3–5	Laparotomy	Sevoflurane	Morphine	s.c.	3	–	–	–	Yes
Thomas et al. (87)	Laparotomy + Morphine i.t.	Group	3–5	Laparotomy	Sevoflurane	Morphine	i.t.	0.2	–	–	–	Yes
Waite et al. (88)	Buprenorphine 15 min prior to surgery 0.01	Group	4	Laparotomy	Isoflurane	Buprenorphine	s.c.	0.01	–	–	–	Yes
Waite et al. (88)	Buprenorphine 15 min prior to surgery 0.025	Group	4	Laparotomy	Isoflurane	Buprenorphine	s.c.	0.025	–	–	–	Yes
Waite et al. (88)	Buprenorphine intraoperatively 0.01	Group	4	Laparotomy	Isoflurane	Buprenorphine	s.c.	0.01	–	–	–	Yes
Waite et al. (88)	Buprenorphine intraoperatively 0.025	Group	4	Laparotomy	Isoflurane	Buprenorphine	s.c.	0.025	–	–	–	Yes
Waite et al. (88)	Buprenorphine intraoperatively 0.05	Group	4	Laparotomy	Isoflurane	Buprenorphine	s.c.	0.05	–	–	–	Yes
Waite et al. (88)	Carprofen 15 min prior to surgery 5	Group	4	Laparotomy	Isoflurane	Carprofen	s.c.	5	–	–	–	Yes
Waite et al. (88)	Carprofen 15 min prior to surgery 15	Group	4	Laparotomy	Isoflurane	Carprofen	s.c.	15	–	–	–	Yes
Waite et al. (88)	Carprofen intraoperatively 5	Group	4	Laparotomy	Isoflurane	Carprofen	s.c.	5	–	–	–	Yes
Waite et al. (88)	Carprofen intraoperatively 10	Group	4	Laparotomy	Isoflurane	Carprofen	s.c.	10	–	–	–	Yes
Waite et al. (88)	Carprofen intraoperatively 15	Group	4	Laparotomy	Isoflurane	Carprofen	s.c.	15	–	–	–	Yes
Waite et al. (88)	Carprofen intraoperatively 25	Group	4	Laparotomy	Isoflurane	Carprofen	s.c.	25	–	–	–	Yes
Waite et al. (88)	Acetaminophen 15 min prior to surgery 50	Group	4	Laparotomy	Isoflurane	Acetaminophen	s.c.	50	–	–	–	Yes
Waite et al. (88)	Acetaminophen 15 min prior to surgery 100	Group	4	Laparotomy	Isoflurane	Acetaminophen	s.c.	100	–	–	–	Yes
Waite et al. (88)	Acetaminophen intraoperatively 25	Group	4	Laparotomy	Isoflurane	Acetaminophen	s.c.	25	–	–	–	Yes
Waite et al. (88)	Acetaminophen intraoperatively 50	Group	4	Laparotomy	Isoflurane	Acetaminophen	s.c.	50	–	–	–	Yes
Waite et al. (88)	Acetaminophen intraoperatively 100	Group	4	Laparotomy	Isoflurane	Acetaminophen	s.c.	100	–	–	–	Yes
Waite et al. (88)	Ibuprofen 15 min prior to surgery 15	Group	4	Laparotomy	Isoflurane	Ibuprofen	s.c.	15	–	–	–	Yes
Waite et al. (88)	Ibuprofen 15 min prior to surgery 30	Group	4	Laparotomy	Isoflurane	Ibuprofen	s.c.	30	–	–	–	Yes
Waite et al. (88)	Ibuprofen intraoperatively 5	Group	4	Laparotomy	Isoflurane	Ibuprofen	s.c.	5	–	–	–	Yes
Waite et al. (88)	Ibuprofen intraoperatively 15	Group	4	Laparotomy	Isoflurane	Ibuprofen	s.c.	15	–	–	–	Yes
Waite et al. (88)	Ibuprofen intraoperatively 30	Group	4	Laparotomy	Isoflurane	Ibuprofen	s.c.	30	–	–	–	Yes
Waite et al. (88)	Ketoprofen 15 min prior to surgery 10	Group	4	Laparotomy	Isoflurane	Ketoprofen	s.c.	10	–	–	–	Yes
Waite et al. (88)	Ketoprofen 15 min prior to surgery 25	Group	4	Laparotomy	Isoflurane	Ketoprofen	s.c.	25	–	–	–	Yes
Waite et al. (88)	Ketoprofen intraoperatively 5	Group	4	Laparotomy	Isoflurane	Ketoprofen	s.c.	5	–	–	–	Yes
Waite et al. (88)	Ketoprofen intraoperatively 10	Group	4	Laparotomy	Isoflurane	Ketoprofen	s.c.	10	–	–	–	Yes
Waite et al. (88)	Ketoprofen intraoperatively 15	Group	4	Laparotomy	Isoflurane	Ketoprofen	s.c.	15	–	–	–	Yes
Waite et al. (88)	Ketoprofen intraoperatively 25	Group	4	Laparotomy	Isoflurane	Ketoprofen	s.c.	25	–	–	–	Yes
Yamanaka et al. (89)	Surgery + LPS + DEX + Atipamezol (Antagonist)	Group	2	Plantar paw incision	Isoflurane	–	–	–	–	–	–	–
Yousef et al. (90)	Nerve autografting group	Single before surgery	1	Neurosurgery	Tiletamine and zolazepam	–	–	–	–	–	–	–
**Nest-Building mice**												
Abdelrahman et al. (96)	Pancreatic cancer model	Single before surgery	1	Laparotomy	Isoflurane	Carprofen	s.c.	5	–	–	–	–
Arras et al. (97)^+^	Laparotomy + carprofen	Single before surgery	1	Laparotomy	Sevoflurane	Carprofen	s.c.	5	–	–	–	–
Arras et al. (97)^+^	Laparotomy + flunixin	Single before surgery	1	Laparotomy	Sevoflurane	Flunixin	s.c.	5	–	–	–	–
Beninson et al. (98)	Carprofen	Single from surgery on	1				5	–	–	–	–	No
Beninson et al. (98)	Robenacoxib	Single from surgery on	1	laparotomy	isoflurane	Laparotomy	Isoflurane	Carprofen	–	–	–	No
Cesarovic et al. (99)^+^	Surgery + anesthesia + analgesia	Single before surgery	1	Laparotomy	Sevoflurane	Carprofen	s.c.	5	–	–	–	–
Falkenberg et al. (100)	Catheterization common carotid artery + ligation	Single before surgery	1	Vascular surgery	Isoflurane	Buprenorphine	Oral (nute paste)	1	Buprenorphine	s.c.	0.1	–
Gallo et al. (58)	Nest material + surgery + analgesia	Single before surgery	1	Vascular surgery	Ketamine and xylazine	Buprenorphine	s.c.	0.05	–	–	–	No
Herndon et al. (101)	CLP + buprenorphine hydrochloride (Bup HCI)	Group	5	Laparotomy	Isoflurane	Buprenorphine	s.c.	0.1	–	–	–	–
Herndon et al. (101)	CLP + buprenorphine sustained-release (Bup SR)	Group	5	Laparotomy	Isoflurane	Buprenorphine SR	s.c.	1	–	–	–	–
Jirkof et al. (102)	Surgery + anesthesia + analgesia + single housing	Single from surgery on	1	Laparotomy	Sevoflurane	Carprofen	s.c.	5	–	–	–	–
Jirkof et al. (102)	Surgery + anesthesia + analgesia + pair housing	Group	2	Laparotomy	Sevoflurane	Carprofen	s.c.	5	–	–	–	–
Jirkof et al. (27)	Surgery + anesthesia + low dose analgesia	Single before surgery	1	Laparotomy	Sevoflurane	Carprofen	s.c.	5	–	–	–	No
Jirkof et al. (27)	Surgery + anesthesia + high dose analgesia	Single before surgery	1	Laparotomy	Sevoflurane	Carprofen	s.c.	50	–	–	–	No
Jirkof et al. (103)	Surgery + anesthesia + analgesia + familiar cage after surgery during burrowing	Group	3 to 6	Laparotomy	Sevoflurane	Carprofen	s.c.	5	–	–	–	–
Jirkof et al. (61)	Anesthesia and surgery with T:P in the drinking water	Single before surgery	1	Laparotomy	Sevoflurane	Tramadol	Oral (drinking supply)	–	Paracetamol	Oral (drinking supply)	–	No
Kendall et al. (104)	Laparotomy + Bup-HCI	Single	1	Laparotomy	Isoflurane	Buprenorphine	s.c.	0.1	–	–	–	Yes
Kendall et al. (104)	Laparotomy + Bup-SR	Single	1	Laparotomy	Isoflurane	Buprenorphine SR	s.c.	0.6	–	–	–	Yes
Kumstel et al. (105)	Transmitter implantation	Single before surgery	1	Laparotomy	Isoflurane	Carprofen	s.c.	5	Metamizole	Oral (drinking supply)	–	–
Oliver et al. (106)	Pair housed, anesthesia + buprenorphine, washout, surgery + buprenorphine	Group	2	Laparotomy	Isoflurane	Buprenorphine	s.c.	0.1	–	–	–	–
Oliver et al. (106)	Pair housed, anesthesia + carprofen, washout, surgery + carprofen	Group	2	Laparotomy	Isoflurane	Carprofen	Oral (drinking supply)	30	–	–	–	–
Oliver et al. (106)	Pair housed, anesthesia + multimodal, washout, surgery + multimodal	Group	2	Laparotomy	Isoflurane	Carprofen	Oral (drinking supply)	30	Buprenorphine	s.c.	–	Yes
Oliver et al. (106)	Single housed, anesthesia + buprenorphine, washout, surgery + buprenorphine	Single before surgery	1	Laparotomy	Isoflurane	Buprenorphine	s.c.	0.1	–	–	–	–
Oliver et al. (106)	Single housed, anesthesia + carprofen, washout, surgery + carprofen	Single before surgery	1	Laparotomy	Isoflurane	Carprofen	Oral (drinking supply)	30	–	–	–	–
Oliver et al. (106)	Single housed, anesthesia + multimodal, washout, surgery + multimodal	Single before surgery	1	Laparotomy	Isoflurane	Carprofen	Oral (drinking supply)	30	Buprenorphine	s.c.	–	Yes
Oliver et al. (106)	Single + nest during baseline, anesthesia + buprenorphine, washout, surgery + buprenorphine	Single from surgery on	1	Laparotomy	Isoflurane	Buprenorphine	s.c.	0.1	–	–	–	–
Oliver et al. (106)	Single + nest during baseline, anesthesia + carprofen, washout, surgery + carprofen	Single from surgery on	1	Laparotomy	Isoflurane	Carprofen	Oral (drinking supply)	30	–	–	–	–
Oliver et al. (106)	Single + nest during baseline, anesthesia + multimodal, washout, surgery + multimodal	Single from surgery on	1	Laparotomy	Isoflurane	Carprofen	Oral (drinking supply)	30	Buprenorphine	s.c.	–	Yes
Pham et al. (107)^+^	Enriched + surgery	Group	3	Laparotomy	Isoflurane	Ibuprofen	Oral (drinking supply)	–	–	–	–	–
Pham et al. (107)^+^	Enriched + surgery	Single from surgery on	1	Laparotomy	Isoflurane	Ibuprofen	Oral (drinking supply)	–	–	–	–	–
Robinson-Junker et al. (108)	Predictable sleep disruption + control (saline)	Single before surgery	1	Biopsy	Isoflurane	Lidocaine	Topical	–	–	s.c.	–	Yes
Robinson-Junker et al. (108)	Predictable sleep disruption + analgesia	Single before surgery	1	Biopsy	Isoflurane	Lidocaine	Topical	–	Carprofen	s.c.	10	Yes
Rock et al. (28)	Carotid artery injury	Single before surgery	1	Vascular surgery	Isoflurane	Buprenorphine	s.c.	0.05	–	–	–	–
Rock et al. (28)	Carotid artery injury	Group	2–5	Vascular surgery	Isoflurane	Buprenorphine	s.c.	0.05	–	–	–	–
Staib-Lasarzik et al. (109)	Subarachnoid hemorrhage + buprenorphine	–	–	Vascular surgery	Isoflurane	Buprenorphine	s.c.	0.1	–	–	–	–
Staib-Lasarzik et al. (109)	Subarachnoid hemorrhage + carprofen	–	–	Vascular surgery	Isoflurane	Carprofen	s.c.	5	–	–	–	–
Staib-Lasarzik et al. (109)	Subarachnoid hemorrhage + meloxicam	–	–	Vascular surgery	Isoflurane	Meloxicam	s.c.	1	–	–	–	–
Staib-Lasarzik et al. (109)	Controlled cortical impact + buprenorphine	–	–	Craniotomy	Isoflurane	Buprenorphine	s.c.	0.1	–	–	–	–
Staib-Lasarzik et al. (109)	Controlled cortical impact + carprofen	–	–	Craniotomy	Isoflurane	Carprofen	s.c.	5	–	–	–	–
Staib-Lasarzik et al. (109)	Controlled cortical impact + meloxicam	–	–	Craniotomy	Isoflurane	Meloxicam	s.c.	1	–	–	–	–
Van-Loo et al. (110)	Laparotomy + single housed	Single before surgery	1	Laparotomy	Isoflurane	Carprofen	s.c.	5	–	–	–	–
Van-Loo et al. (110)	Laparotomy + housed with non-operated cage mate	Group	2	Laparotomy	Isoflurane	Carprofen	s.c.	5	–	–	–	–
Yuan et al. (111)^+^	MCAO 20 min	Single from surgery on	1	Vascular surgery	Isoflurane	–	–	–	–	–	–	–
**Nest building rats**												
Möller et al. (112)	Craniotomy	Single before surgery	1	Craniotomy	Chloral hydrate	Meloxicam	s.c.	1	Bupivacaine	s.c.	–	–
**Burrowing mice**												
Abdelrahman et al. (96)	Pancreatic cancer model	Single before surgery	1	Laparotomy	Isoflurane	Carprofen	s.c.	5	–	–	–	–
Evangelista-Vaz et al. (56)	Surgery + anesthesia + tramadol injection + drinking supply	Group	4–8	Laparotomy	Sevoflurane	Tramadol	s.c.	25	Tramadol	Oral (drinking supply)	25	No
Jirkof et al. (8)	Surgery + anesthesia + analgesia	Single before surgery	1	Laparotomy	Sevoflurane	Carprofen	s.c.	5	–	–	–	Yes
Jirkof et al. (102)	Surgery + anesthesia + analgesia + single housing	Single from surgery on	1	Laparotomy	Sevoflurane	Carprofen	s.c.	5	–	–	–	Yes
Jirkof et al. (102)	Surgery + anesthesia + analgesia + pair housing	Group	2	Laparotomy	Sevoflurane	Carprofen	s.c.	5	–	–	–	Yes
Jirkof et al. (103)	Surgery + anesthesia + analgesia + familiar cage after surgery during burrowing	Group	3–6	Laparotomy	Sevoflurane	Carprofen	s.c.	5	–	–	–	–
Jirkof et al. (59)	OPT1 (surgery + anesthesia + T1)	Single before surgery	1	Laparotomy	Sevoflurane	Buprenorphine	s.c.	0.1	–	–	–	Yes
Jirkof et al. (59)	OPSB (surgery + anesthesia + SB)	Single before surgery	1	Laparotomy	Sevoflurane	Buprenorphine SR	s.c.	2.2	–	–	–	Yes
Jirkof et al. (61)	Anesthesia and surgery with T:P in the drinking water	Single before surgery	1	Laparotomy	Sevoflurane	Tramadol	Oral (drinking supply)	–	Paracetamol	Oral (drinking supply)	–	Yes
Kumstel et al. (105)	Transmitter implantation	Single before surgery	1	Laparotomy	Isoflurane	Carprofen	s.c.	5	Metamizole	Oral (drinking supply)	–	–
Shepherd et al. (113)	SNI + Gabapentin	Group	5	Neurosurgery	Isoflurane	Gapapentin	i.p.	10	–	–	–	Yes
Shi et al. (114)	Disc degeneration disease	–	–	Neurosurgery	Isoflurane	–	–	–	–	–	–	–
**Burrowing rats**												
Andrews et al. (26)	TNT + gabapentin low dose (SD rats)	Group	4	Neurosurgery	Isoflurane	Gapapentin	s.c.	30	–	–	–	Yes
Andrews et al. (26)	TNT + gabapentin high dose (SD rats)	Group	4	Neurosurgery	Isoflurane	Gapapentin	s.c.	100	–	–	–	Yes
Andrews et al. (26)	L5 SNT –> neurosurgery + strain2 (Wistar rats)	Group	4	Neurosurgery	Isoflurane	–	–	–	–	–	–	–
Deseure and Hans (31)	IoN ligation	–	–	Neurosurgery	Pentobarbital	–	–	–	–	–	–	–
Georgieva et al. (115)	Acute DHA treatment	Group	2–3	Laminectomy	Isoflurane	Buprenorphine	s.c.	0.3	Carprofen	s.c.	50	–
Katri et al. (116)	Meniscectomy + Naproxen	Group	3–4	Meniscectomy	Isoflurane	Xylocain	Topical	–	Carprofen	–	–	–
Lau et al. (117)	SNI	Group	2	Neurosurgery	Ketamine and xylazine	–	–	–	–	–	–	–
Möller et al. (112)	Craniotomy	Single before surgery	1	Craniotomy	Chloral hydrate	Meloxicam	s.c.	1	Bupivacaine	s.c.	–	–
Muralidharan et al. (118)	CCI sciatic nerve	Group	2–3	Neurosurgery	Isoflurane	–	–	–	–	–	–	–

–not reported. Alphabetical characters in the Study ID indicate animal groups within individual studies [first column of the tables; e.g., Cho et al. (54)]. ^*^in comparison with control group. To test the effect of analgesia, groups with surgical intervention and analgesia were compared with a control group, which was defined as a group with surgical intervention without analgesia. ^+^References were identified during screening of reference lists. SR, sustained-release.

### Post-surgical analysis of nest building activity and performance

**Figure 3:**
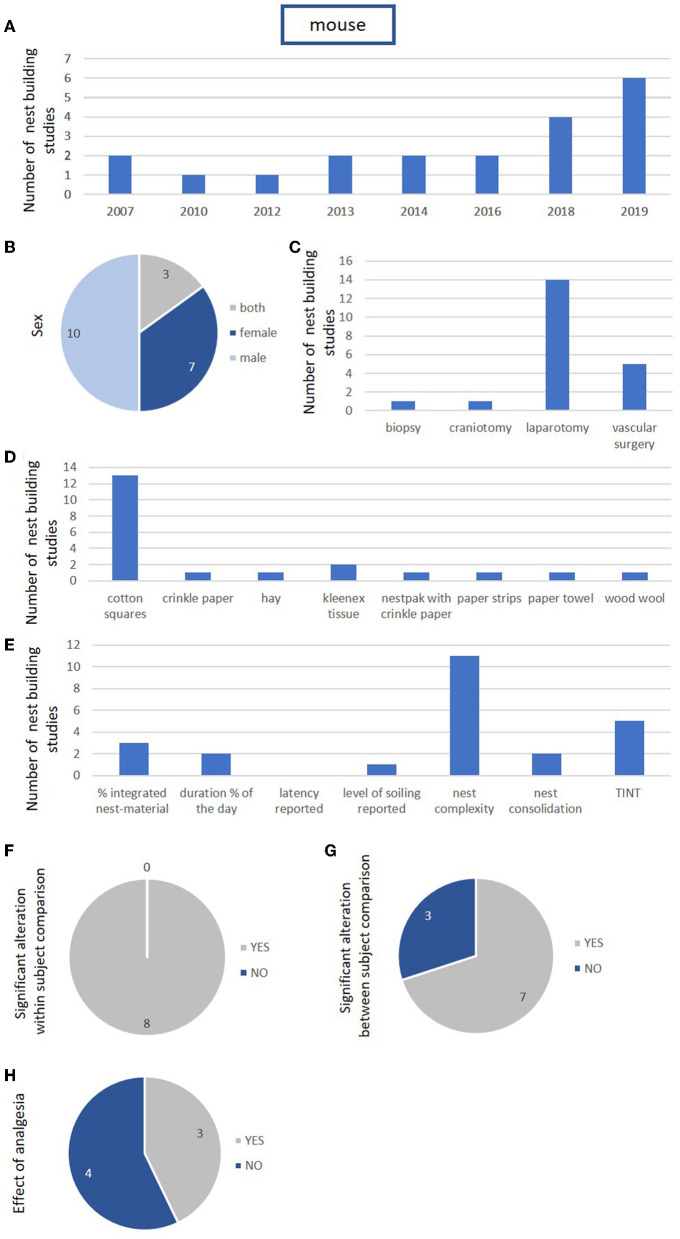
Study characteristics, animal characteristics, intervention characteristics, and outcome characteristics for nest building in mice (k = 20 studies). **(A)** Number of published studies using nest building for the assessment of post-surgical pain in mice during the last decade. X-axis represents years. **(B)** Number of included nest building studies using females, males, and both sexes. **(C)** Type of intervention in mice. **(D)** Used nest building material in mice. **(E)** Reported nest building parameters in mice. **(F)** Studies describing within-subject comparison in mice. The within-subject comparison revealed an influence of the surgical intervention in all studies. **(G)** Studies describing between-subject comparison in mice. **(H)** Studies describing an analysis of the impact of an analgetic drug in comparison with a control group.

Screening identified 20 studies published between 2007 and 2020 that explored nest-building activity and performance in the post-surgical phase of interventions in mice (27, 28, 58, 61, 96–111). For rats, we only identified one study from Germany published in 2018 that assessed the impact of surgery on nest building in female Sprague Dawley rats following craniotomy (112). Concerning mouse studies, the countries of origin of the first author comprise different European countries and the United States (Table 6). The number of mouse studies with analysis of nest building following surgery increased slightly toward the end of the studied decade (Figure 3A).

**Table 6:** Study and animal characteristics for nest building in mice (k = 20 studies) and rats (k = 1 study).

**Study ID**	**Year of publication**	**Country of origin first author**	**Strain**	**Breeder**	**Sex**	**Age arrival [weeks]**	**Body weight on arrival [g]**	**Body weight on evaluation [g]**
**Mice**								
Abdelrahman et al. (96)	2019	Germany	C57BL/6J	–	Male	–	–	–
Arras et al. (97)^+^	2007	Switzerland	NMRI	Harlan	Male	4	–	40–54
Beninson et al. (98)	2018	USA	CFW	Charles River	Male	5–8	–	28.9
Cesarovic et al. (99)^+^	2014	Switzerland	C57BL/6J	In house-breeding	Both	6–8	–	–
Cesarovic et al. (99)^+^	2014	Switzerland	DBA/2J	In house-breeding	Both	6–8		
Falkenberg et al. (100)	2019	Denmark	NMRI	Taconic	Male	6	–	–
Gallo et al. (58)	2019	USA	Crl:CD1(ICR)	Charles River	Male	8–9	–	–
Herndon et al. (101)	2016	USA	C57BL/6	Charles River	Male	–	24–28	–
Jirkof et al. (102)	2012	Switzerland	C57BL/6J	In-House breeding	Female	6–8	–	–
Jirkof et al. (27)	2013	Switzerland	C57BL/6J	In House-breeding	Female	6–8	–	–
Jirkof et al. (103)	2013	Switzerland	C57BL/6J	In-House breeding	Female	6–8	–	–
Jirkof et al. (61)	2018	Switzerland	C57BL/6J	Charles River	Female	6–8	–	–
Kendall et al. (104)	2016	USA	Crl:CD1(ICR)	Charles River	Female	8–10	20–30	–
Kumstel et al. (105)	2019	Germany	C57BL/6J	–	Male	13–15	–	–
Oliver et al. (106)	2018	USA	Crl:CD1(ICR)	Charles River	Both	7–12	–	–
Oliver et al. (106)	2018	USA	C57BL/6	Charles River	Both	6–12		
Pham et al. (107)^+^	2010	Sweden	C57BL/6	B&K Universal AB	Female	–	21–25	–
Robinson-Junker et al. (108)	2019	USA	C57BL/6N	Charles River	Both	6	–	–
Rock et al. (28)	2014	USA	–	Jackson laboratories	Male	16	–	–
Staib-Lasarzik et al. (109)	2019	Germany	C57BL/6N	Charles River	Male	–	18–23	–
Van-Loo et al. (110)	2007	Netherlands	C57BL/6J	Charles River	Female	9	–	–
Yuan et al. (111)^+^	2018	USA	C57BL/6	Charles River	Male	10–12	–	–
**Rats**								
Möller et al. (112)	2018	Germany	SD	Envigo	Female	–	200–224	–

–not reported. Alphabetical characters in the Study ID indicate animal groups within individual studies [first column of the tables; e.g., Cesarovic et al. (99)]. ^+^References were identified during screening of reference lists.

The list of mouse studies included three studies that completed an analysis in both sexes, ten studies that focused on male mice, and seven studies that focused on female mice (Table 6, Figure 3B).

Information about the mouse strain used could be extracted from the majority of publications. The analysis revealed a predominance of studies in C57BL/6J mice with a total number of eight studies. In four publications, it was not specified which C57BL/6 substrain was used, i.e., J or N. For further mouse strains reported in the identified publications, the number of studies per strain amounted to one to two (Table 6).

Considering a frequently applied habituation phase of 1–2 weeks following arrival, age on arrival indicated that the vast majority of studies (15/20) in mice were completed in young adult mice. The body weight at the time of the surgical intervention or nest building assessment was only reported in two studies. Younger mice (<6 weeks) were ordered (age 4 or 5–8 weeks at arrival) from the commercial breeder in only two studies (97, 98). Arras et al. (97) reported that animals underwent laparotomy at 16 weeks of age to assess post-surgical pain 3 days after surgery. In the study by Beninson et al. (98), the age of the animals at the time of surgery and nest building assessment was not reported.

In all of the mouse studies, animals were no older than 15 weeks on arrival (Table 6).

Concerning the type of surgical intervention, laparotomy was the most frequently performed procedure in mice, conducted in 14 studies. Other interventions in mice comprised vascular surgery, biopsy, and craniotomy (Figure 3C).

Cotton squares represented the most frequently used nesting material in mouse studies (13/20 studies). Alternative materials comprised wood wool, crinkle paper, paper strips, kleenex tissues, paper towels, hay, and nestpak with crinkle paper (Table 7, Figure 3D). Only five mouse studies reported the amount of material offered. An image-based evaluation proved to be the exception, reported in just two publications (Table 7).

**Table 7:** Outcome characteristics for nest building in mice (k = 20 studies) and rats (k = 1 study).

**Study ID**	**Nest material**	**Size (cm) material**	**Amount (g) material**	**Image based evaluation**	**Time of evaluation in relation to dark**	**Baseline**	**Which scoring system was used?**	**Significant alteration of nest building parameters**		**Evaluated parameter/comments**
								**Within-subject comparison**	**Between-subject comparison**	
**Mice**										
Abdelrahman et al. (96)	Cotton squares	5 × 5	–	–	Light phase	Yes	0–6	Yes	–	Nest complexity
Arras et al. (97)^+^	Hay	–	18–20	–	–	Yes	0–1	–	–	Nest complexity, descriptive evaluation
Beninson et al. (98)	Cotton squares	–	–	–	–	Yes	1–5	Yes	No	Nest consolidation
Cesarovic et al. (99)^+^	Cotton squares	5 × 5	–	Yes	Light phase	–	–	–	–	–
Falkenberg et al. (100)	Wood wool	–	6	Yes	Light phase	Yes	0–5	–	Yes	Nest complexity; % integrated material
Gallo et al. (58)	Crinkle paper	–	10	–	Light phase	Yes	0–5	Yes	Yes	Nest complexity, TINT
Herndon et al. (101)	Cotton squares	–	3	–	Light phase	Yes	–	–	No	Nest complexity, TINT
Jirkof et al. (102)	Cotton squares	5 × 5	–	–	Light phase	–	–	–	–	Duration % of the day, level of soiling
Jirkof et al. (27)	Cotton squares	5 × 5	–	–	Light phase	Yes	0–5	Yes	Yes	Nest complexity
Jirkof et al. (103)	Cotton squares	5 × 5	–	–	Light phase	–	–	–	–	Duration % of the day
Jirkof et al. (61)	Cotton squares	5 × 5	–	–	Light phase	–	0–5	–	Yes	Nest complexity
Kendall et al. (104)	Paper strips	–	–	–	–	Yes	–	–	Yes	TINT; % integrated material
Kumstel et al. (105)	Cotton squares	5 × 5	–	–	Light phase	–	1–6	Yes	–	Nest complexity
Oliver et al. (106)	Cotton squares	–	–	–	Both	Yes	1–5	Yes	–	Nest consolidation
Oliver et al. (106)	Cotton squares, enviropak	–	–	–	Both	Yes	1–5	Yes	–	Nest consolidation
Pham et al. (107)^+^	Kleenex tissue	–	–	–	–	–	–	–	No	Nest complexity
Robinson-Junker et al. (108)	Cotton squares	–	–	–	Light phase	Yes	–	Yes	Yes	TINT
Rock et al. (28)	Cotton squares	–	–	–	Light phase	Yes	–	Yes	–	TINT
Staib-Lasarzik et al. (109)	Paper towel	–	–	–	–	Yes	0–2	–	–	Nest complexity, visual assessment score
Van-Loo et al. (110)	Kleenex tissue	–	–	–	–	Yes	1–4	–	–	Nest complexity, descriptive evaluation
Yuan et al. (111)^+^	Cotton squares	5 × 5	2,5	–	–	–	–	–	Yes	% integrated material
**Rats**										
Möller et al. (112)	Crinkle paper	–	14	Yes	Light phase	Yes	0–3	–	No	Latency, nest complexity, level of soiling

–not reported. Alphabetical characters in the Study ID indicate animal groups within individual studies [first column of the tables; Oliver et al. (106)]. ^+^References were identified during screening of reference lists.

While seven publications failed to provide information about the time of the day for assessment, the majority of studies (12/20) focused on the light phase and only one study assessed the activity during the light and dark phase (Table 7). The type of assessment and parameters varied across the mouse studies with application of the TINT (= time-to-integrate to nest test) in five studies, assessment of nest consolidation in two studies, of nest complexity in eleven studies, of % integrated material in three studies, and duration of nest building activity (% of the day) in two studies. An additional analysis of the level of soiling was only described in one study. The analysis of more than one nest building parameter was rather an exception (Table 7, Figure 3E).

Baseline data were collected in 13 of the 20 mouse studies. A comparison with baseline levels (within-subject design) was described in eight of the mouse studies. An impact of the surgical intervention on nest building activity or performance based on a between-subject design was assessed in 10 mouse studies. The within-subject comparison revealed an effect in 8 and the between-subject comparison revealed an effect in seven mouse studies (Table 7, Figures 3F,G).

An impact of a single analgetic or combination of analgetic drugs on nest-building activity or performance in comparison with the control group was analyzed in seven of the 20 mouse studies. The analysis revealed an impact in three studies (Table 5, Figure 3H).

### Post-surgical analysis of burrowing activity and performance

Screening identified 10 mouse (8, 56, 59, 61, 96, 102, 103, 105, 113, 114) and 7 rat studies (26, 31, 112, 115–118) published between 2010 and 2019 that explored burrowing activity in the post-surgical phase of interventions.

The countries of origin of the first author comprised Switzerland, Germany, the United States, and China for the mouse studies, and the United Kingdom, the United States, and four different European countries for the rat studies (Table 8).

**Table 8:** Study and animal characteristics for burrowing mice (k = 10 studies) and rats (k = 7 studies).

**Study ID**	**Year of publication**	**Country of origin first author**	**Journal**	**Breeder**	**Sex**	**Age arrival [weeks]**	**Body weight on arrival [g]**	**Body weight on evaluation [g]**
**Mice**								
Abdelrahman et al. (96)	2019	Germany	C57BL/6J	–	Male	–	–	–
Evangelista-Vaz et al. (56)	2018	Switzerland	C57BL/6J	Charles River	Female	6–8	18–22	–
Jirkof et al. (8)	2010	Switzerland	C57BL/6J	In-House breeding facility	Both	6–8	–	–
Jirkof et al. (102)	2012	Switzerland	C57BL/6J	In-House breeding facility	Female	6–8	–	–
Jirkof et al. (103)	2013	Switzerland	C57BL/6J	In-House breeding facility	Female	6–8	–	–
Jirkof et al. (59)	2015	Switzerland	C57BL/6J	Charles River	Female	6–8	–	–
Jirkof et al. (61)	2018	Switzerland	C57BL/6J	Charles River	Female	6–8	–	–
Kumstel et al. (105)	2019	Germany	C57BL/6J	–	Male	13–15	–	–
Shepherd et al. (113)	2018	USA	C57BL/6J	Jackson laboratories	Both	8–14	–	–
Shepherd et al. (113)	2018	USA	FVB/NJ	Jackson laboratories	Both	8–14	–	–
Shi et al. (114)	2018	China	C57BL/6J	In-House breeding facility	Female	–	20–25	–
**Rats**								
Andrews et al. (26)	2011	UK	SD	Charles River Ltd UK	–	–	175–200	–
Andrews et al. (26)	2011	UK	Wistar	B&K Universal Ltd UK	–	–	175–200	–
Deseure and Hans (31)	2018	Belgium	SD	Charles River	Male	–	225–250	–
Georgieva et al. (115)	2019	UK	Wistar	Charles River	Male	–	180–200	–
Katri et al. (116)	2019	Denmark	Lewis	Envigo	Female	6–8	170–200	–
Lau et al. (117)	2013	Canada	SD	Charles River	Male	8	–	200–230
Möller et al. (112)	2018	Germany	SD	Envigo	Female	–	200–224	–
Muralidharan et al. (118)	2016	Australia	SD	Animal resources center	Male	–	180–200	200–250

–not reported. Alphabetical characters in the Study ID indicate animal groups within individual studies [first column of the tables; e.g., Shepherd et al. (113)]. ^+^References were identified during screening of reference lists.

**Figure 4:**
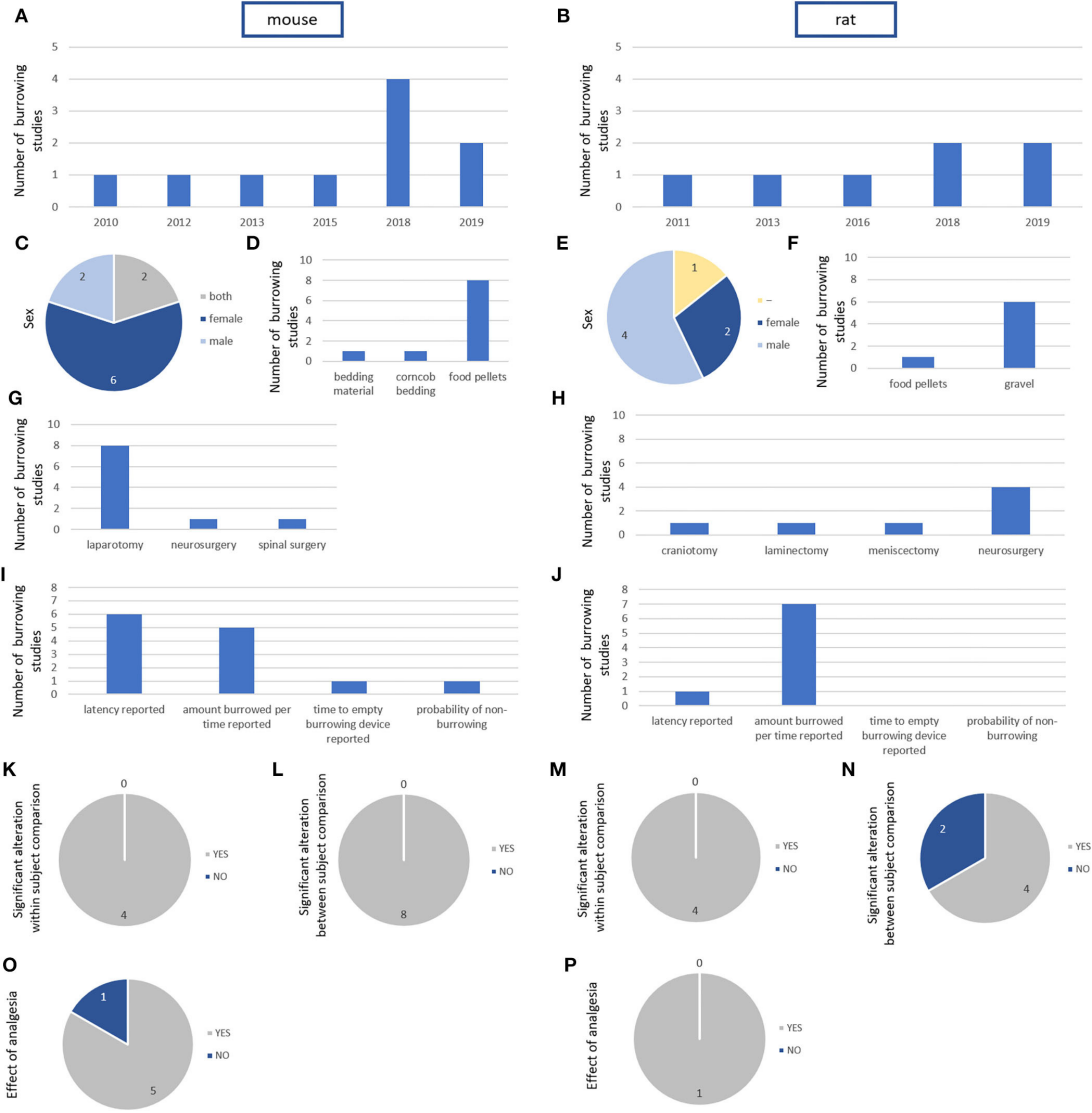
Study characteristics, animal characteristics, and intervention characteristics for burrowing in mice (k = 10 studies) and rats (k = 7 studies). **(A,B)** Number of published studies using burrowing for the assessment of post-surgical-pain in mice **(A)** and rats **(B)** during the last decade. X-axis represents years. **(C,E)** Number of burrowing studies in mice **(C)** and rats **(E)** using females, males, and both sexes. **(D,F)** Used burrowing material in mice **(D)** and rats **(F)**. **(G,H)** Type of intervention in mice **(G)** and rats **(H)**. **(I,J)** Reported burrowing parameter in mice **(I)** and rats **(J)**. **(K,M)** Studies describing within-subject comparison in mice **(K)** and rats **(M)**. The within-subject comparison revealed an influence of the surgical intervention in all mouse and all rat studies. **(L,N)** Studies describing between-subject comparison in mice **(L)** and rats **(N)**. The between-subject comparison revealed an influence of the surgical intervention in all mouse studies. **(O,P)** Studies describing an analysis of the impact of an analgetic drug in comparison with a control group in mice **(O)** and rats **(P)**. The analysis revealed an influence in all rat studies.

The first mouse study assessing burrowing activity in the post-surgical phase was published in 2010 and the first rat study in 2011. In mice, a recent trend for an increase in the number of studies/year was identified (Table 8, Figures 4A,B).

While two studies assessed burrowing in mice of both sexes, six studies focused on female mice and two on male mice. Among the publications reporting burrowing data from rats, one failed to report the sex of the animals, four focused on male rats and two on female rats (Table 8, Figures 4C,E).

Information about the mouse and rat strains used could be extracted from all the publications. In all mouse studies, the experiments were conducted in C57BL/6J mice. One study additionally used FVB/NJ mice (113). The strains used in the rat studies comprised Sprague Dawley rats (five studies), Wistar rats (two studies), and Lewis rats (one study; Table 8).

Considering a frequently applied habituation phase of 1–2 weeks following arrival, age or body weight on arrival indicated that the vast majority of studies (16/17) were completed in young adult or adult mice and rats. The body weight at the time of the surgical intervention or burrowing assessment was not reported in most studies (15/17; Table 8).

Food pellets represented the most frequently used burrowing material in mouse studies (8/10 studies). Only two studies used bedding material as an alternative for the burrowing test in mice. The amount of burrowing material offered varied greatly across the mouse studies, ranging from 50 to 201 g. Almost all studies in rats offered gravel (2,000 or 2,500 g) as the burrowing material except for one study using food pellets (1,000 g, Table 9, Figures 4D,F).

**Table 9:** Outcome characteristics for burrowing mice (k = 10 studies) and rats (k = 7 studies).

**Study ID**	**Burrowing material**	**Amount burrowing material (g)**	**Duration of test (min)**	**Video/Image based evaluation**	**Time of evaluation in relation to dark**	**Baseline**	**Significant alteration of burrowing parameters**		**Evaluated parameter**
							**Within-subject comparison**	**Between-subject comparison**	
**Mice**									
Abdelrahman et al. (96)	Food pellets	199–201	120	–	Light phase	Yes	Yes	–	Amount burrowed per time
Evangelista-Vaz et al. (56)	Food pellets	–	720	Yes	Dark phase	–	–	Yes	Latency
Jirkof et al. (8)	Food pellets	138–142	120	Yes	Light phase	Yes	Yes	Yes	Latency; Amount burrowed per time; Time to empty burrowing device
Jirkof et al. (102)	Food pellets	138–142	360–1,440	Yes	Light phase	–	–	Yes	Latency
Jirkof et al. (103)	Food pellets	138–142	360–1,440	Yes	Light phase	–	–	Yes	Latency
Jirkof et al. (59)	Food pellets	138–142	720	Yes	–	–	–	Yes	Latency
Jirkof et al. (61)	Food pellets	–	1,440	Yes	Both	–	–	Yes	Latency, Probability of non-burrowing
Kumstel et al. (105)	Food pellets	200	120	–	Light phase	–	Yes	–	Amount burrowed per time
Shepherd et al. (113)	Corncob bedding	50	15	–	–	Yes	Yes	Yes	Amount burrowed per time
Shi et al. (114)	Bedding material	200	10	–	–	Yes	–	Yes	Amount burrowed per time
**Rats**									
Andrews et al. (26)	Gravel	2,500	60	–	–	Yes	Yes	Yes	Amount burrowed per time
Andrews et al. (26)	Gravel	2,500	120	–	–	Yes	Yes	Yes	Amount burrowed per time
Deseure and Hans (31)	Food pellets	1,000	240	–	–	Yes	Yes	Yes	Amount burrowed per time
Georgieva et al. (115)	Gravel	2,500	120	–	Light phase	Yes	–	Yes	Amount burrowed per time
Lau et al. (117)	Gravel	2,500	60	–	Dark phase	Yes	–	Yes	Amount burrowed per time
Katri et al. (116)	Gravel	–	–	–	–	Yes	–	No	Amount burrowed per time
Möller et al. (112)	Gravel	2,500	60	Yes	Light phase	Yes	–	No	Latency, Amount burrowed per time
Muralidharan et al. (118)	Gravel	2,000	60	No	Light phase	Yes	Yes	–	Amount burrowed per time

–not reported. Alphabetical characters in the Study ID indicate animal groups within individual studies [first column of the tables; e.g., Andrews et al. (26)]. ^+^References were identified during screening of reference lists.

Concerning the type of surgical intervention, laparotomy proved to be the predominant procedure in mice (k = 8). Only two mouse studies focused on a neurosurgical procedure. In rats, the list of interventions comprised neurosurgery, craniotomy, laminectomy, and meniscectomy (Figures 4G,H).

Latency to start burrowing was the most frequently assessed parameter in the mouse studies with six studies reporting the respective data. The amount burrowed per time was analyzed in five mouse studies. In these studies, the duration of the test ranged from 10 to 120 min. Further parameters analyzed in just one study each were time to empty the burrowing device and probability of non-burrowing. All rat studies assessed the amount burrowed per time. The duration of the test varied ranging from 60 to 240 min. Only one rat study reported an additional analysis of the latency to start burrowing activity (Table 9, Figures 4I,J).

Concerning time-of-day for the analysis of burrowing in the mouse studies, three publications failed to provide the respective information, five studies conducted the assessment during the light phase, one study analyzed the activity during the dark phase, and one study during the light and dark phases. For rats, three publications failed to provide the respective information, three studies focused on an assessment during the light phase, and one study on an assessment during the dark phase (Table 9).

Baseline data were collected in 4 of the 10 mouse studies and all of the rat studies (7/7).

A comparison with baseline levels (within-subject design) was described in four of the mouse studies and three of the rat studies. The within-subject comparison revealed an influence in all four of these mouse studies and all three of these rat studies (Figures 4K,M). An impact of the surgical intervention on burrowing activity or performance compared to a separate control group (between-subject design) was assessed in eight of the mouse studies and six of the rat studies. The between-subject comparison revealed an influence in all eight of these mouse studies and four of these six rat studies (Table 9, Figures 4L,N).

An impact of a single analgetic or combination of analgetic drugs on burrowing activity or performance in comparison with the control group was analyzed in 6 of 10 mouse studies. The analysis revealed an impact in five of these studies (Figure 4O). In rats, the impact of analgetic or different analgetic drugs on burrowing activity or performance was assessed in only one study where an effect was present (Table 5, Figure 4P).

### Anesthesia and analgesia

In both species, inhalational anesthesia was most frequently applied with the use of isoflurane most common, followed by the use of sevoflurane. Further types of anesthetic drugs and drug combinations comprised ketamine/xylazine in mice as well as ketamine, ketamine/xylazine, tiletamine/zolazepam, pentobarbital, and chloral hydrate in rats (Table 5).

**Figure 5:**
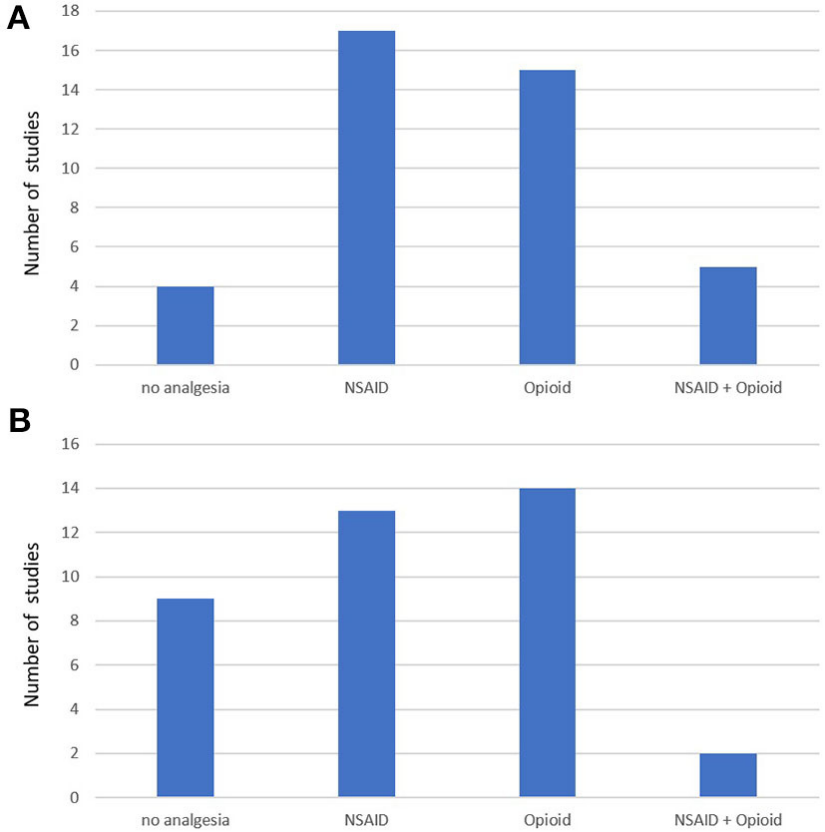
Type of analgesia in mice (k = 39 studies) and rats (k = 36 studies) combined for grimace, nest building and burrowing. **(A)** Type of analgesia used in mice. **(B)** Type of analgesia used in rats.

Perioperative analgetic drugs were administered in the vast majority of studies (62/75). However, almost all studies used a monotherapeutic approach with the application of only one type of analgetic drug (Figure 5). An opioid was administered in 38% of the mouse studies (15/39) and 39% of the rat studies (14/36). The list of opioids comprised buprenorphine and tramadol in mice and buprenorphine, nalbuphine, morphine, fentanyl, and tramadol in rats. Use of a non-steroidal anti-inflammatory (NSAID) drug was reported in 44% of the mouse studies (17/39) and 36% of the rat studies (13/36). The list of NSAIDs included carprofen, ibuprofen, ketoprofen, meloxicam, flunixin, robenacoxib in mice and carprofen, (dex)ibuprofen, ketoprofen, and meloxicam in rats (Table 5).

A multimodal approach was used in 13% of the mouse studies (5/39) and 6% of the rat studies (2/36). Local anesthetic drugs were only applied in 5% of the mouse (2/39) and 33% of the rat studies (12/36) with the use of the following drugs: lidocaine, bupivacaine in mice and lidocaine, ropivacaine, (levo)bupivacaine, xylocaine in rats (Table 5).

Moreover, the analgetic-antipyretic acetaminophen was administered in two mouse studies (61, 64) and one rat study (88). Further drugs used included metamizole and gabapentin in mice and thalidomide and gabapentin in rats (Table 5).

### Application of measures to control the risk of bias and reporting quality

**Figure 6:**
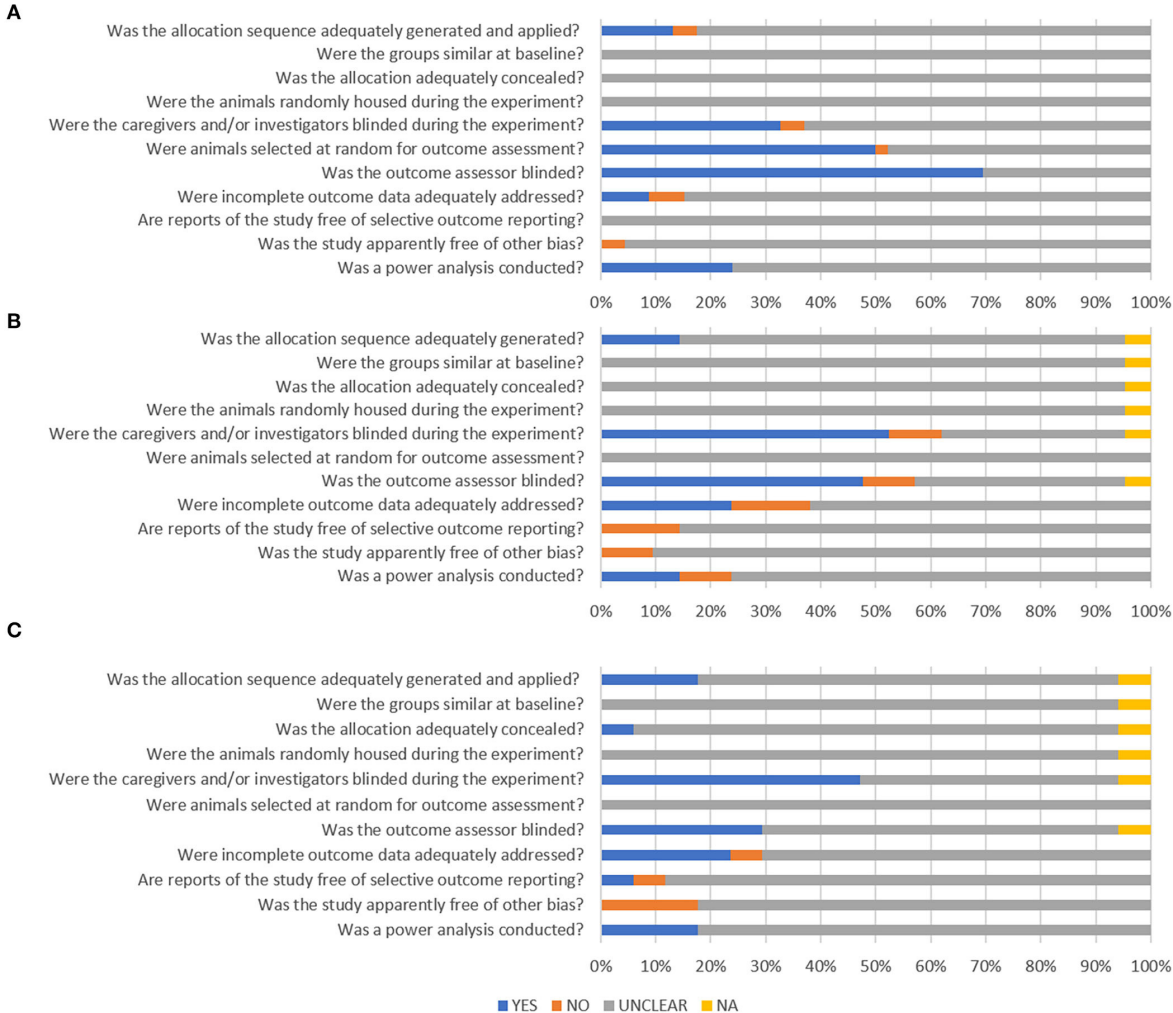
Risk of bias assessment for grimace scale **(A)**, nest building **(B)**, and burrowing **(C)**. Mice and rats were pooled together per pain parameter. YES, scored a low risk of bias; NO, scored a high risk of bias; UNCLEAR, scored an unclear risk of bias; NA, not applicable; RoB item was not applicable as only one group was used in the study.

For each included study and each pain parameter, we have assessed the risk of bias (Figure 6).

Regarding selection bias, information about baseline characteristics was missing in 99% and information about the procedures for allocation concealment was missing in 96% of all studies identified for the different pain parameters. Moreover, details about the randomization approach for sequence generation were only provided in 14% of the publications (Figure 6).

Assessment of performance bias focused on random housing and blinding. While information about the distribution of animals and cages in the animal facility was missing in 98% of the publications, blinding of caregivers and/or investigators from knowledge about the intervention was reported in 40% of the publications (Figure 6). While not formally analyzed, during data extraction, we noticed that details about the staff members that remained blinded were rarely provided.

Randomization of animals for testing or of videos and images for outcome assessment was reported in 50% of the studies assessing grimace scales, in none of the publications assessing nest building, and in none of the studies assessing burrowing.

Details about the blinding of assessors were provided in 70% of the grimace scale studies, 48% of the nest building studies, and 29% of the burrowing studies.

Incomplete reporting of attrition and exclusion was evident in the majority of the studies. The completeness of outcome data remained unclear in 76% of all studies identified for the different pain parameters. Moreover, in 94% of the publications, it remained unclear whether the reports were free of selective outcome reporting. Other types of bias were identified in 8% of the studies. It was not reported whether a power analysis was conducted in 77% of the studies (Figure 6).

## Discussion

The reliable and robust monitoring and scoring of pain in laboratory rodents is an important basis for optimized perisurgical pain management, which always needs to be adjusted to the animal characteristics and the specific intervention. Moreover, for the selection of the analgetic regimen, a potential impact on scientific readout parameters needs to be considered (5). Considering the multitude of influencing factors, it is obvious that there are no universally valid analgetic concepts applicable to all experimental interventions. This, together with the general tendency of prey animals to hide signs of pain and suffering, implies the particular relevance of sensitive approaches to monitor pain and to check the success of pain management approaches.

Selected grimace scale AUs and behavioral parameters are considered as the key parameters for comprehensive composite evaluation schemes (56, 59, 61, 68). The relevance of these parameters is underscored by the fact that alterations in physiological and biochemical markers such as heart rate, respiratory rate, blood pressure, core temperature, and corticosterone are not specific for pain states.

This systematic review aimed to determine the use and evidence-base of three tests applied in the context of post-surgical pain assessment in mice and rats.

Traditional narrative reviews carry the risk of containing only a subset of the literature relevant to the topic that is known to the author conducting the review and thus may contain a bias based on the author's opinion and network (119, 120). In contrast, systematic reviews aim to analyze all relevant literature on a predefined research question, generate new data, and ideally summarize the results with a meta-analysis (120). We here present a systematic review based on a comprehensive search. The methodology was transparently described in a previously posted protocol. Furthermore, the selection of studies based on pre-defined eligibility criteria for inclusion was systematically performed in two screening phases. The quality of the studies was assessed using SYRCLE's risk of bias tool. The generated data from the included studies were systematically presented and analyzed in tabular form. A meta-analysis was not performed because the heterogeneity in experimental designs and outcomes between the included studies was considered to be too high. Although our search aimed to be fully comprehensive, reference list screening identified 10 additional papers. Part of this was due to some authors only using the relatively specific term “home cage behavior” instead of the specific test in their titles and abstracts or database indexing for the behavioral tests being suboptimal, but we also missed the relevant term “laminectomy” in the search string for surgery. We highly recommend adding this term to future searches for surgeries. The total number of studies identified amounted to 74. While the number of studies identified per parameter and year tended to increase slightly toward the second half of the last decade, the overall numbers are too low to conclude a trend for an increased application of the parameters. In rats, the RGS, which has initially been described by Sotocinal et al. (34), was the most frequently applied parameter. In mice, the highest numbers of studies were identified for MGS and nest building applied in the post-surgical phase.

Our findings with grimace scales being among the more frequently used are in line with recent reviews stating that facial expressions are widely used as a pain assessment parameter in laboratory rodents (41, 46). In this context, it needs to be emphasized that the analysis of the countries of origin of the first author indicated that we are far from a widespread global application of the parameters of interest.

As recently discussed by Turner et al. (10), the majority of commonly applied measures of pain are indirect and thus only provide an approximation of the actual pain state. Along this line, it has been emphasized that reliable pain detection in humans as well as animals requires multidimensional composite assessment schemes (10, 14, 121). Respective composite schemes can, for instance, combine a behavioral-based scale with fecal corticosterone metabolite levels (122). In this context, it seems unfortunate that only a small number of studies identified in the current review combined two or three of the parameters of interest. Thus, it is impossible to conclude about the relative or added informative value of the three parameters, which were in the focus of this review.

Grimace scales build on the fact that an interaction of neural pathways between peripheral receptors and efferent facial motor neurons results in changes in facial expression. These changes seem to be evolutionarily preserved as a “pain face” can, for instance, trigger social attention, protection, and care including maternal care (123–126). The initial development and characterization of the MGS comprised an assessment in various assays with an activation of the nociceptive system including the following surgical procedures: laparotomy, chronic constriction injury, and spared nerve injury conducted in female and male Crl:CD1(ICR) mice (33). The findings suggested that MGS can help to monitor visceral and somatic pain following laparotomy, but fail to detect neuropathic pain. Since this first report, a further seventeen studies have analyzed the MGS during the post-surgical phase. The majority of studies used young adult C57BL/6J or Crl:CD1*(*ICR) mice. Thus, it is evident that the application of the MGS following surgery has so far not been sufficiently assessed in other mouse strains as well as in younger and older mice. As strain and age may well impact the head shape, an impact on the different AUs of the MGS seems likely.

A comparable situation is evident for the RGS with a focus on young adult Wistar or Sprague Dawley rats in the majority of studies. While there was a lack of studies exploring the RGS in younger animals, some studies were identified that focused on aged rats (72, 77, 80, 82, 87, 93, 95). In the initial study, Sotocinal et al. (34) have already reported an RGS-based detection of post-laparotomy pain in young Wistar rats. Considering the since conducted studies, there is an obvious need to further evaluate the RGS in younger rats and in rat strains other than Wistar and Sprague Dawley rats. Concerning the type of surgery, the list of mouse and rat studies indicates that a higher level of experience has been reached for laparotomy since the first publications in 2010 and 2011. For the majority of other interventions, the number of studies identified per species did not exceed three studies.

Considering the technical aspects, the fact that the majority of studies report an image- or video-based analysis of MGS and RGS provides evidence that many scientists have tried to limit the risk of bias associated with a direct evaluation of grimace scores related to the observer's presence (10). In an earlier study, live scores proved to be lower than retrospective video-based scores (127). In this context, another important factor is that live or video-based scoring can better consider the changing facial expression so that transient blinking will not result in altered scores. Our data suggest that more research seems to be necessary to validate the different scoring approaches with live vs. video- vs. image-based scoring systems by direct comparison.

A recent systematic review focused on grimace scales in non-human mammals has already intensely studied the level of evidence for measurement properties of various grimace scales reporting a high level of evidence for MGS and RGS (46). Our analysis focusing on post-surgical pain revealed that the majority of studies analyzed all AUs of the MGS and RGS. However, some of the mouse studies did not consider whisker scores and a very small number of studies focused on orbital tightening and ear position only. This finding is in line with difficulties reported for a reliable assessment of whisker position (10, 25, 84), resulting in the decision to disregard this action unit. In this context, it is of interest that a recent study ranked the relative importance of MGS AUs based on two different mathematical approaches (128). While orbital tightening was identified as the best parameter, whisker change and nose bulge were the worst performing variables in this study focusing on pain responses to intraperitoneal CCl_4_ injection (128). The authors concluded that the findings suggest that the MGS can be simplified; however, they also emphasized that a model-specific assessment of the informative value of AUs might be necessary (128).

Several studies have assessed the ability to demonstrate the impact of the surgical intervention based on a within- and/or a between-subject comparison confirming the suitability and sensitivity of grimace scales to detect post-surgical pain following different procedures. An impact of the surgical intervention was observed in all within-subject comparisons for mice and rats, and in most studies including a between-subject comparison.

Further evidence comes from studies which explored an effect of the analgetic regimen based on a reduction in MGS and RGS scores, which was observed in the vast majority of studies.

While these data support the application of grimace scales for pain assessment, it needs to be considered that the evaluator's experience, knowledge, and training can have a tremendous impact on the assessment of subjective parameters such as facial expression and behavior (40, 129, 130).

The fact that we were only able to identify one rat study that explored nest-building activity and quality following a surgical intervention (112) seems to reflect difficulties to detect a reliable construction of complex nests in rats (131, 132). In apparent contrast, mice seem to be characterized by a higher intrinsic level of motivation for nest construction. This may be related to species differences in thermoregulation related to the ratio between body surface and body weight, resulting in a higher need for shelter and protection from weather and climate influences in wildlife mice as compared to wildlife rats.

Thus, it is not surprising that the validity of nest-building activity as an animal welfare and pain assessment parameter has been explored more intensely in mice (9, 10). Arras et al. (97) provided one of the first reports describing a post-surgical reduction in nest quality following laparotomy. Follow-up studies from the same group further tested the assessment of nest building for detection of post-surgical pain in mice of both sexes and different strains (27, 133).

Considering the publications identified by our systematic review, the majority of nest-building studies have focused on young adult mice with C57BL/6J as the most frequently used mouse strain and laparotomy as the most frequently applied surgical intervention. Thus, there are obvious gaps in knowledge concerning the post-surgical analysis of nest building in younger and aged mice, mouse strains other than C57BL/6J, and for surgical procedures other than laparotomy.

Concerning the methodological aspects, we identified a high variance in the type of parameters assessed ranging from time-to-integrate to nest (28, 58, 101, 104, 108), nest consolidation (98, 106), nest complexity (27, 58, 61, 96, 97, 100, 101, 105, 107, 109, 110), % integrated material (100, 104, 111), and % time spent nest building (102, 103). While it is of interest that efforts have been made to develop and explore different readout parameters, this of course limits the total level of evidence for the different parameters. Thus, it is recommended to directly compare the different nest building parameters in standardized approaches to provide information about potential differences in sensitivity, inter-rater and intra-rater reliabilities, and robustness.

An influence of the surgical intervention on nest building was reported by all studies with a within-subject design and the majority of studies with a between-subject comparison. While these data may support nest building as a parameter for post-surgical pain assessment, the low number of studies with a respective assessment and the heterogeneity in study design and parameters analyzed needs to be considered. Moreover, conflicting results were evident in studies assessing the impact of an analgetic. As already discussed by Jirkof (9), it still remains unclear whether the failure to detect an analgetic effect is due to residual pain resulting from insufficient pain control, high sensitivity of the parameters to low levels of residual pain control or a limited informative value of nest building as a pain parameter. In this context, it needs to be considered that nest-building activity is also compromised by impairments other than pain, including influences related to experimental infection or models of systemic inflammation, neurodegenerative, and psychiatric disorders (9, 134–138). Thus, based on the current state of knowledge, it is recommended that nest building such as other pain assessment parameters should only be applied as one parameter of a composite pain measurement scheme and under controlled environmental conditions.

Both mice and rats exhibit a high level of intrinsic motivation for burrowing behavior. A detrimental impact of post-laparotomy pain in mice has initially been described by Jirkof et al. (8). This study and follow-up studies from the same group (56, 59, 61) have not only sparked the interest of laboratory animal scientists but also of companies and academic groups engaged in development and assessment of novel analgetic drug candidates. This resulted in efforts to also assess burrowing as a potential pain assessment parameter in rats (139–141).

The total number of publications with the assessment of burrowing in the post-surgical phase in mice and rats was rather low, indicating that the parameter has not yet been well-characterized for post-surgical monitoring. Conclusions about general evidence are limited by the fact that the majority of studies focused on young adult or adult C57BL/6J mice and Sprague Dawley rats, and that the majority of studies in mice analyzed post-laparotomy pain. Thus, there is an apparent lack or paucity of knowledge concerning the application in younger and aged mice and rats, in other strains, and following different procedures.

Concerning the technical aspects, our systematic review confirms previous narrative reviews stating that food pellets and gravel are the predominant burrowing materials offered to mice and rats (9, 10). In this context, it is of interest that Wodarski et al. (139) have reported that the test was more sensitive when a material with a smaller particle size was offered to rats. This finding is not reflected by common approaches applied in rats with the continued use of gravel.

When comparing the readout parameters, species-specific differences in study design became evident with the majority of mouse studies focusing on the latency to start burrowing, and the majority of rat studies focusing on the amount burrowed per time. As further parameters including time to empty the burrowing device were only assessed in some studies, it is again recommended to conduct studies with direct standardized comparison allowing conclusions about the sensitivity and robustness of the different burrowing parameters.

It is of interest that in all mouse studies, both within-subject and between-subject comparisons confirmed the impact of surgical interventions. Furthermore, the effect of an analgetic regimen on burrowing was analyzed in six mouse studies with the majority (5 studies) confirming an impact. Thus, available data so far seem to support the application of burrowing as one parameter for pain assessment in the post-surgical phase.

However, in view of the low number of studies completing a respective analysis along with the failure to demonstrate an impact of the surgical intervention on burrowing performance in some studies, it is evident that more data are required to conclude the value of burrowing as a post-surgical pain parameter in rats.

While the anesthetic and analgetic management was not the focus of our analysis, we additionally extracted information about the drugs used. Interestingly, the vast majority of studies (62/75) identified by our systematic review protocol reported perioperative administration of analgetic drugs with frequent use of either opioids or NSAIDs. However, multimodal approaches were only applied in a small number of studies. In this context, it seems unfortunate that local anesthesia, which can efficaciously block the transduction of nociceptive signaling, is only rarely used in mice (133).

Concerning future directions, it is of particular interest that efforts are made to develop semi-automatic or automatic analysis based on the training and development of machine learning algorithms (142–144). Respective approaches might help to provide robust information and to standardize the scoring-based assessment of parameters such as the grimace scale. In addition, new approaches for imaging-based analysis might provide a basis for home-cage assessment of compromised well-being and pain. Infrared thermal imaging allows to collect information about changes in blood flow, heart rate, and respiratory rate (145–148), which in combination with automatic behavioral tracking provides an excellent basis for continuous monitoring of wellbeing.

Conclusions about the validity of pain parameters assessed in different studies need to take the study quality and risk of bias into consideration. The respective assessment using SYRCLE's risk of bias tool (48) demonstrated that the risk of bias seems to be unclear or high for most included studies.

In conclusion, the number of studies that analyzed grimace scales, nest building, and burrowing in the post-surgical phase in mice or rats is still relatively low. Gaps in knowledge are evident concerning the application of these parameters in different strains, age levels, and following different surgical procedures as well as their combined use. While findings concerning the impact of an intervention and the influence of an analgetic approach seem to be rather consistent for grimace scales, more data are needed for burrowing and nest building. In this context, further analyses are also necessary to directly compare the sensitivity and robustness of different performance parameters that can be applied for nest building and burrowing activities.

## Data availability statement

The raw data supporting the conclusions of this article will be made available by the authors, without undue reservation.

## Author contributions

HP and AB acquired the funding. HP supervised the review. KA wrote the review protocol and developed the search string under supervision from CL, HP, IK, and PJ. Screening was performed by KA, VB, HS, CH, MB, and HK. KS performed the quality check. KA and HP wrote the manuscript with contributions from HS and CL. All authors read and approved the manuscript.

## Funding

This project was supported by the grants of Deutsche Forschungsgemeinschaft (FOR 2591, GZ: PO681/9-1 and 9-2, and BL 953/11-1 and 11-2). The funders had no role in the study design, data collection and analysis, decision to publish, or preparation of the manuscript.

## Conflict of interest

The authors declare that the research was conducted in the absence of any commercial or financial relationships that could be construed as a potential conflict of interest.

## Publisher's note

All claims expressed in this article are solely those of the authors and do not necessarily represent those of their affiliated organizations, or those of the publisher, the editors and the reviewers. Any product that may be evaluated in this article, or claim that may be made by its manufacturer, is not guaranteed or endorsed by the publisher.
