# Supplementary material for: Grimace scale, burrowing, and nest building for the assessment of post-surgical pain in mice and rats—A systematic review
Source: Front Vet Sci. 2022 Oct 6;9:930005. doi: 10.3389/fvets.2022.930005 (PMC9583882; doi:10.3389/fvets.2022.930005)
Supplement: Supplementary file 1 [file Data_Sheet_1.docx]

Supplementary Material

**Supplementary methods 1: Systematic review protocol**


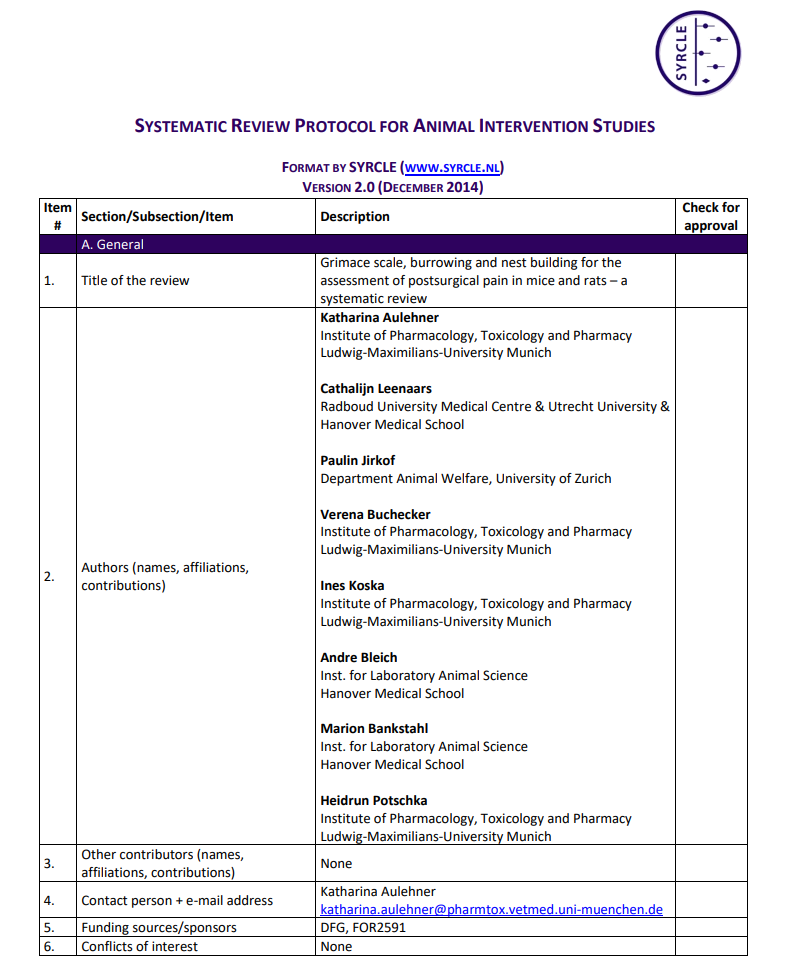


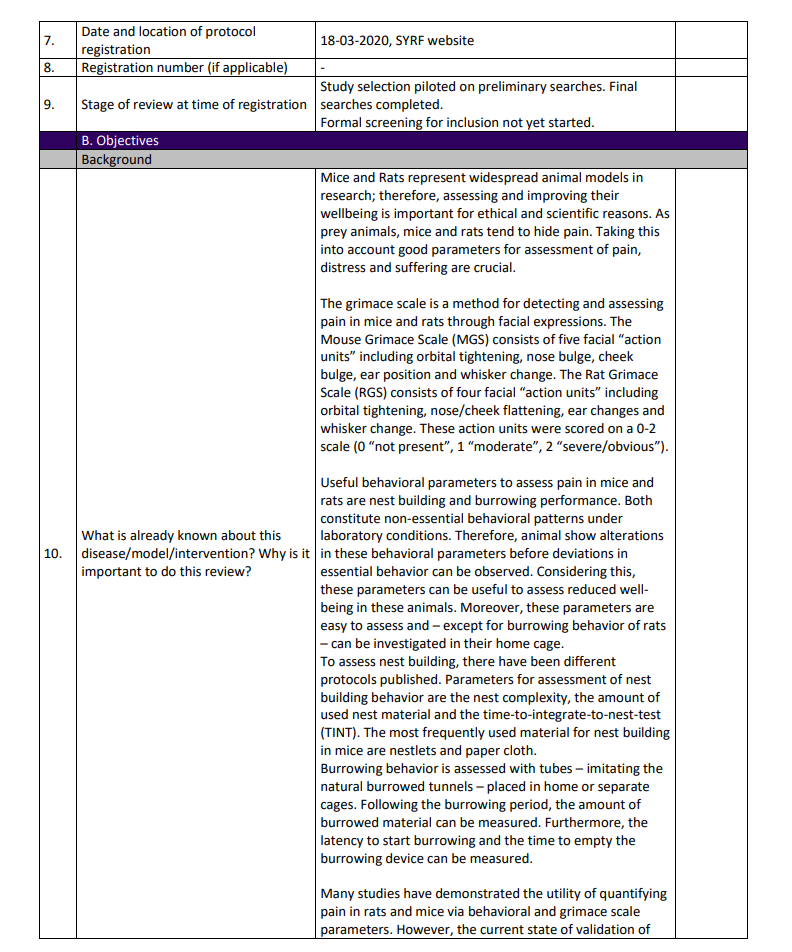


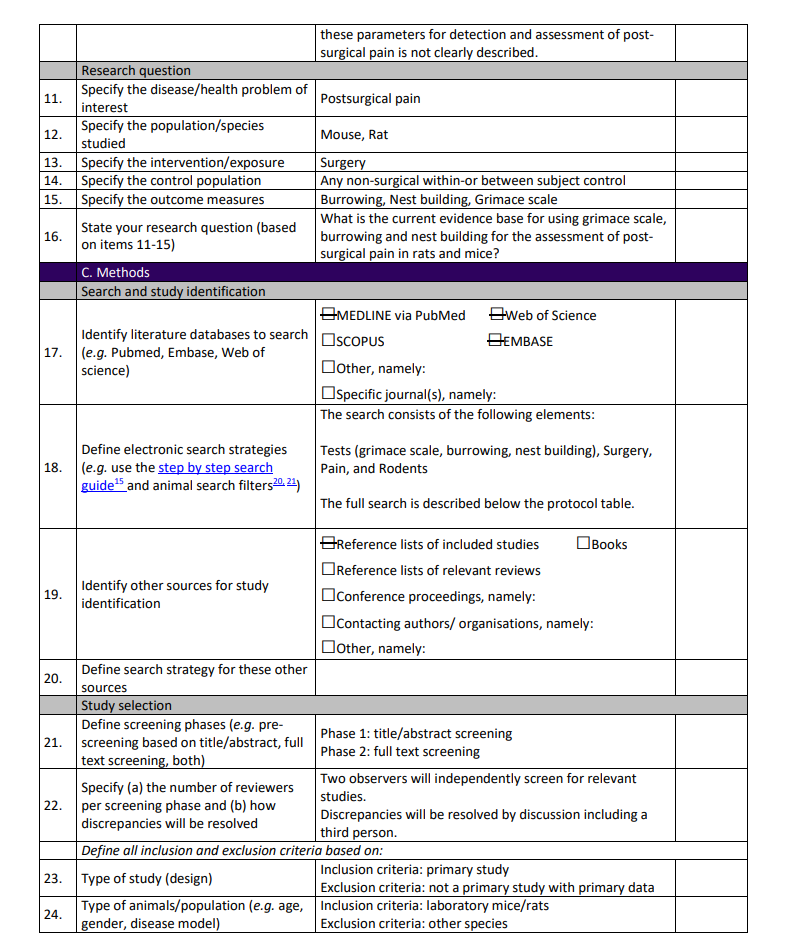


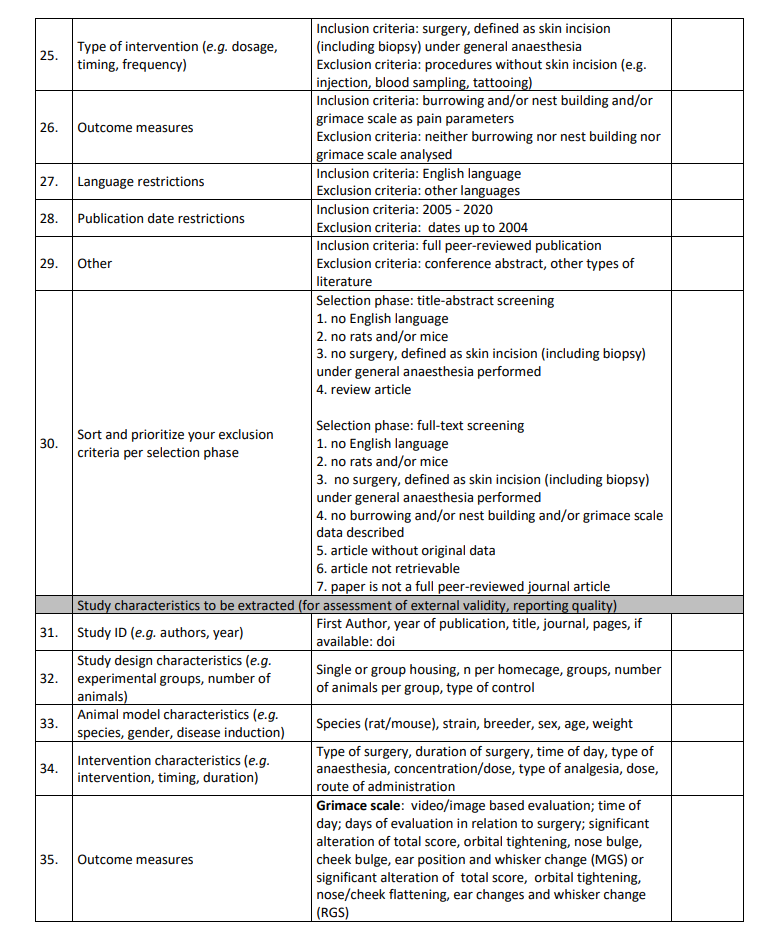


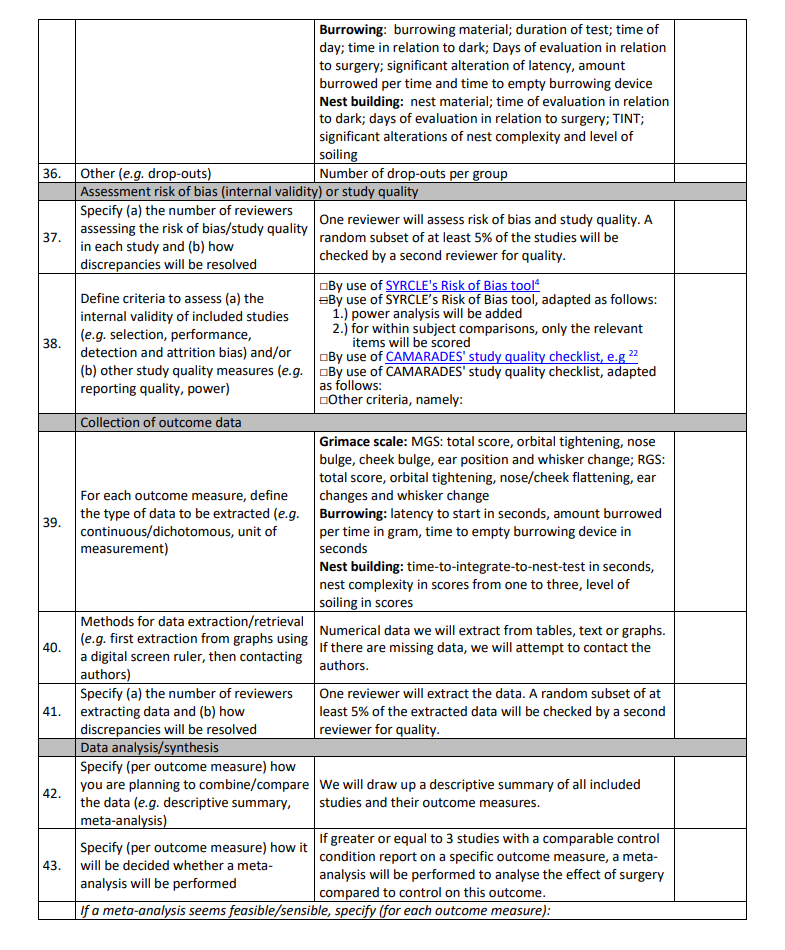


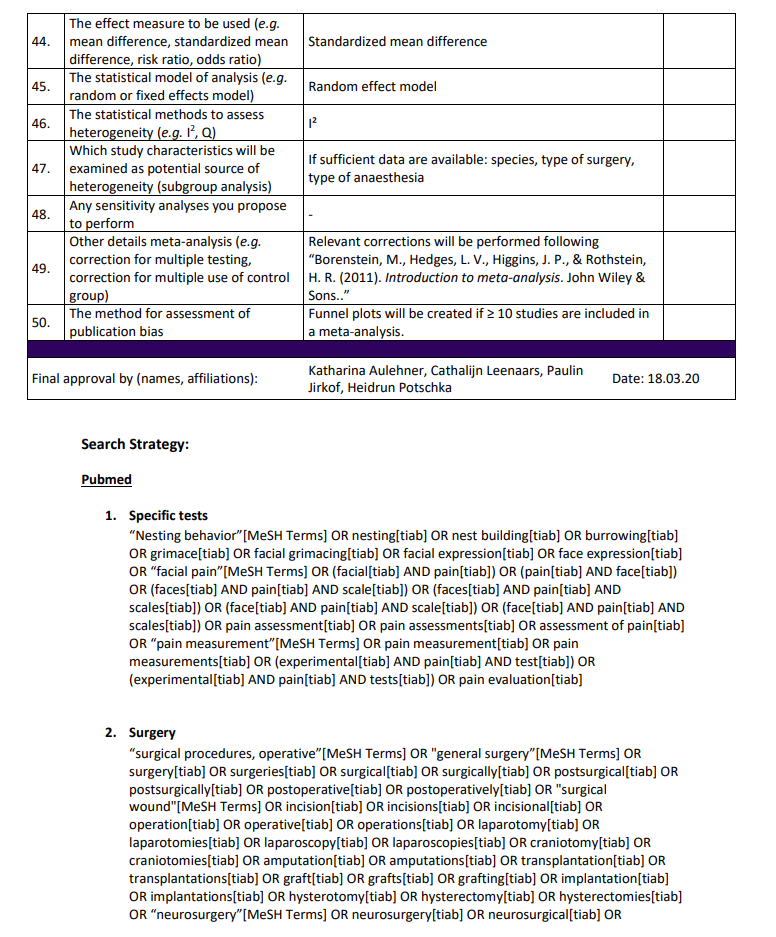


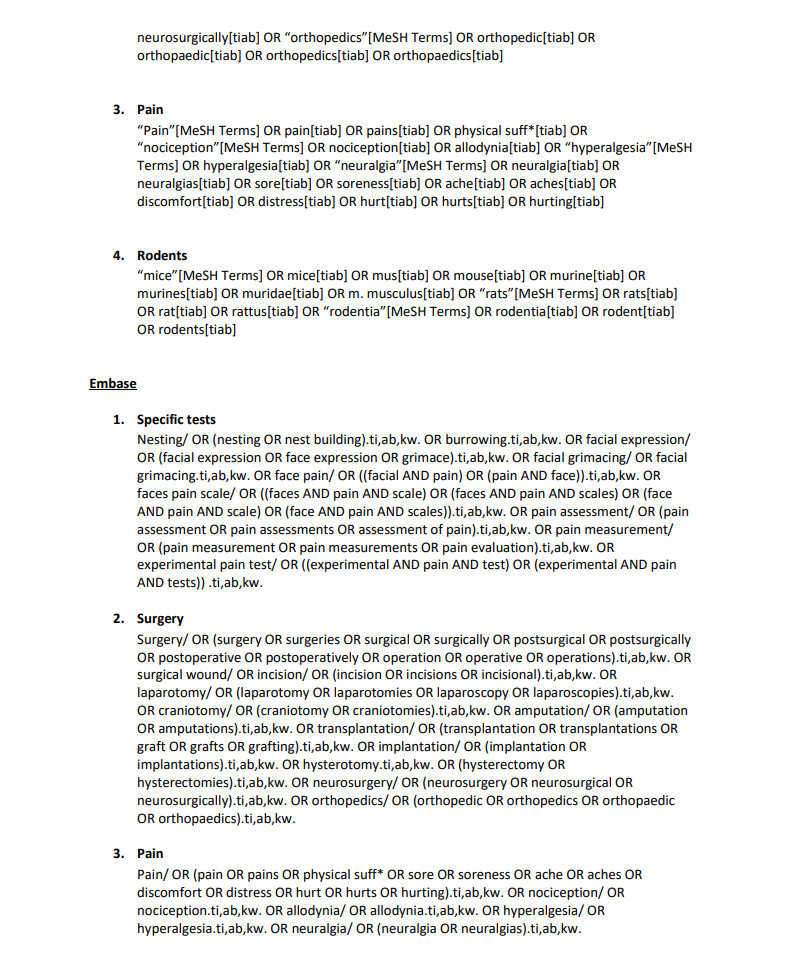


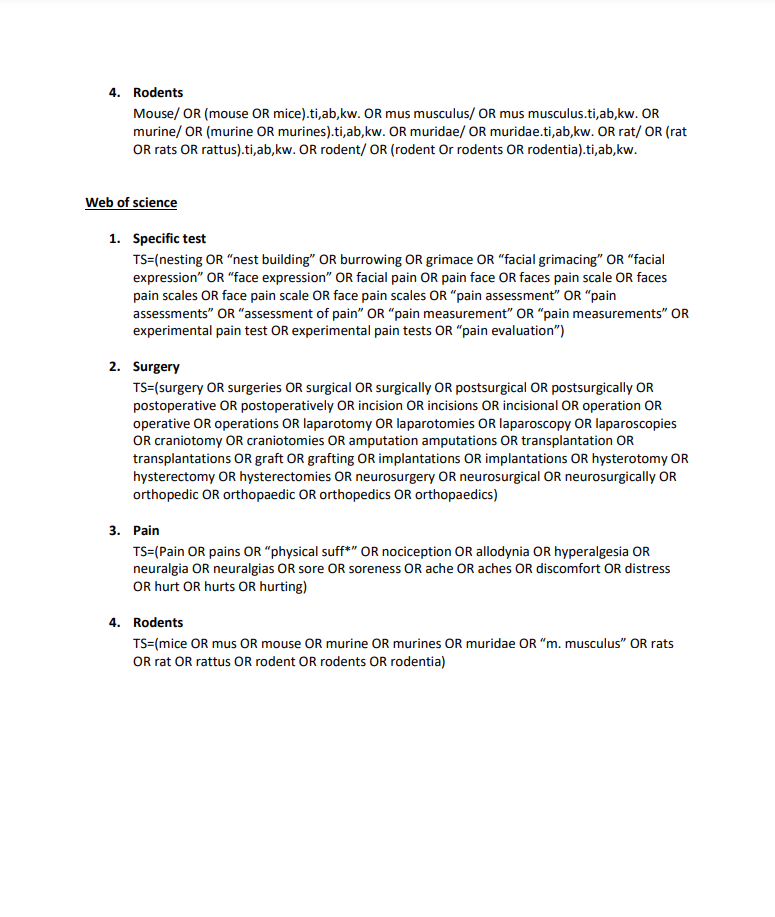


**Supplementary methods 2: PRISMA checklist**

| **Section and Topic** | **Item #** | **Checklist item** | **Location where item is reported** |
| --- | --- | --- | --- |
| **TITLE** | | |  |
| Title | 1 | Identify the report as a systematic review. | p1 |
| **ABSTRACT** | | |  |
| Abstract | 2 | See the PRISMA 2020 for Abstracts checklist. | p2 |
| **INTRODUCTION** | | |  |
| Rationale | 3 | Describe the rationale for the review in the context of existing knowledge. | p4-7 |
| Objectives | 4 | Provide an explicit statement of the objective(s) or question(s) the review addresses. | p7 |
| **METHODS** | | |  |
| Eligibility criteria | 5 | Specify the inclusion and exclusion criteria for the review and how studies were grouped for the syntheses. | p9-10 |
| Information sources | 6 | Specify all databases, registers, websites, organisations, reference lists and other sources searched or consulted to identify studies. Specify the date when each source was last searched or consulted. | p8-9 |
| Search strategy | 7 | Present the full search strategies for all databases, registers and websites, including any filters and limits used. | Suppl. Methods 3 |
| Selection process | 8 | Specify the methods used to decide whether a study met the inclusion criteria of the review, including how many reviewers screened each record and each report retrieved, whether they worked independently, and if applicable, details of automation tools used in the process. | p9-10 |
| Data collection process | 9 | Specify the methods used to collect data from reports, including how many reviewers collected data from each report, whether they worked independently, any processes for obtaining or confirming data from study investigators, and if applicable, details of automation tools used in the process. | p10 |
| Data items | 10a | List and define all outcomes for which data were sought. Specify whether all results that were compatible with each outcome domain in each study were sought (e.g. for all measures, time points, analyses), and if not, the methods used to decide which results to collect. | p10 |
|  | 10b | List and define all other variables for which data were sought (e.g. participant and intervention characteristics, funding sources). Describe any assumptions made about any missing or unclear information. | p10 |
| Study risk of bias assessment | 11 | Specify the methods used to assess risk of bias in the included studies, including details of the tool(s) used, how many reviewers assessed each study and whether they worked independently, and if applicable, details of automation tools used in the process. | p11 |
| Effect measures | 12 | Specify for each outcome the effect measure(s) (e.g. risk ratio, mean difference) used in the synthesis or presentation of results. | NA |
| Synthesis methods | 13a | Describe the processes used to decide which studies were eligible for each synthesis (e.g. tabulating the study intervention characteristics and comparing against the planned groups for each synthesis (item #5)). | NA |
|  | 13b | Describe any methods required to prepare the data for presentation or synthesis, such as handling of missing summary statistics, or data conversions. | NA |
|  | 13c | Describe any methods used to tabulate or visually display results of individual studies and syntheses. | p10 |
|  | 13d | Describe any methods used to synthesize results and provide a rationale for the choice(s). If meta-analysis was performed, describe the model(s), method(s) to identify the presence and extent of statistical heterogeneity, and software package(s) used. | NA |
|  | 13e | Describe any methods used to explore possible causes of heterogeneity among study results (e.g. subgroup analysis, meta-regression). | NA |
|  | 13f | Describe any sensitivity analyses conducted to assess robustness of the synthesized results. | NA |
| Reporting bias assessment | 14 | Describe any methods used to assess risk of bias due to missing results in a synthesis (arising from reporting biases). | NA |
| Certainty assessment | 15 | Describe any methods used to assess certainty (or confidence) in the body of evidence for an outcome. | NA |
| **RESULTS** | | |  |
| Study selection | 16a | Describe the results of the search and selection process, from the number of records identified in the search to the number of studies included in the review, ideally using a flow diagram. | p13, Fig. 1 |
|  | 16b | Cite studies that might appear to meet the inclusion criteria, but which were excluded, and explain why they were excluded. | NA |
| Study characteristics | 17 | Cite each included study and present its characteristics. | p14-21 |
| Risk of bias in studies | 18 | Present assessments of risk of bias for each included study. | p22-23 |
| Results of individual studies | 19 | For all outcomes, present, for each study: (a) summary statistics for each group (where appropriate) and (b) an effect estimate and its precision (e.g. confidence/credible interval), ideally using structured tables or plots. | NA |
| Results of syntheses | 20a | For each synthesis, briefly summarise the characteristics and risk of bias among contributing studies. | NA |
|  | 20b | Present results of all statistical syntheses conducted. If meta-analysis was done, present for each the summary estimate and its precision (e.g. confidence/credible interval) and measures of statistical heterogeneity. If comparing groups, describe the direction of the effect. | NA |
|  | 20c | Present results of all investigations of possible causes of heterogeneity among study results. | NA |
|  | 20d | Present results of all sensitivity analyses conducted to assess the robustness of the synthesized results. | NA |
| Reporting biases | 21 | Present assessments of risk of bias due to missing results (arising from reporting biases) for each synthesis assessed. | NA |
| Certainty of evidence | 22 | Present assessments of certainty (or confidence) in the body of evidence for each outcome assessed. | NA |
| **DISCUSSION** | | |  |
| Discussion | 23a | Provide a general interpretation of the results in the context of other evidence. | p26-31 |
|  | 23b | Discuss any limitations of the evidence included in the review. | p24-25 |
|  | 23c | Discuss any limitations of the review processes used. | p24-25 |
|  | 23d | Discuss implications of the results for practice, policy, and future research. | p31-32 |
| **OTHER INFORMATION** | | |  |
| Registration and protocol | 24a | Provide registration information for the review, including register name and registration number, or state that the review was not registered. | p8 |
|  | 24b | Indicate where the review protocol can be accessed, or state that a protocol was not prepared. | p8 |
|  | 24c | Describe and explain any amendments to information provided at registration or in the protocol. | p12 |
| Support | 25 | Describe sources of financial or non-financial support for the review, and the role of the funders or sponsors in the review. | p33 |
| Competing interests | 26 | Declare any competing interests of review authors. | p32 |
| Availability of data, code and other materials | 27 | Report which of the following are publicly available and where they can be found: template data collection forms; data extracted from included studies; data used for all analyses; analytic code; any other materials used in the review. | p33 |

*From:*  Page MJ, McKenzie JE, Bossuyt PM, Boutron I, Hoffmann TC, Mulrow CD, et al. The PRISMA 2020 statement: an updated guideline for reporting systematic reviews. BMJ 2021;372:n71. doi: 10.1136/bmj.n71

For more information, visit: <http://www.prisma-statement.org/>

**Supplementary methods 3: Search strategy for Pubmed, Embase and WOS**

**Pubmed**

1. **Specific tests**

“Nesting behavior”[MeSH Terms] OR nesting[tiab] OR nest building[tiab] OR burrowing[tiab] OR grimace[tiab] OR facial grimacing[tiab] OR facial expression[tiab] OR face expression[tiab] OR “facial pain”[MeSH Terms] OR (facial[tiab] AND pain[tiab]) OR (pain[tiab] AND face[tiab]) OR (faces[tiab] AND pain[tiab] AND scale[tiab]) OR (faces[tiab] AND pain[tiab] AND scales[tiab]) OR (face[tiab] AND pain[tiab] AND scale[tiab]) OR (face[tiab] AND pain[tiab] AND scales[tiab]) OR pain assessment[tiab] OR pain assessments[tiab] OR assessment of pain[tiab] OR “pain measurement”[MeSH Terms] OR pain measurement[tiab] OR pain measurements[tiab] OR (experimental[tiab] AND pain[tiab] AND test[tiab]) OR (experimental[tiab] AND pain[tiab] AND tests[tiab]) OR pain evaluation[tiab]

1. **Surgery**

“surgical procedures, operative”[MeSH Terms] OR "general surgery”[MeSH Terms] OR surgery[tiab] OR surgeries[tiab] OR surgical[tiab] OR surgically[tiab] OR postsurgical[tiab] OR postsurgically[tiab] OR postoperative[tiab] OR postoperatively[tiab] OR "surgical wound"[MeSH Terms] OR incision[tiab] OR incisions[tiab] OR incisional[tiab] OR operation[tiab] OR operative[tiab] OR operations[tiab] OR laparotomy[tiab] OR laparotomies[tiab] OR laparoscopy[tiab] OR laparoscopies[tiab] OR craniotomy[tiab] OR craniotomies[tiab] OR amputation[tiab] OR amputations[tiab] OR transplantation[tiab] OR transplantations[tiab] OR graft[tiab] OR grafts[tiab] OR grafting[tiab] OR implantation[tiab] OR implantations[tiab] OR hysterotomy[tiab] OR [hysterectomy](https://en.wikipedia.org/wiki/Hysterectomy)[tiab] OR hysterectomies[tiab] OR “neurosurgery”[MeSH Terms] OR neurosurgery[tiab] OR neurosurgical[tiab] OR neurosurgically[tiab] OR “orthopedics”[MeSH Terms] OR orthopedic[tiab] OR orthopaedic[tiab] OR orthopedics[tiab] OR orthopaedics[tiab]

1. **Pain**

“Pain”[MeSH Terms] OR pain[tiab] OR pains[tiab] OR physical suff*[tiab] OR “nociception”[MeSH Terms] OR nociception[tiab] OR allodynia[tiab] OR “hyperalgesia”[MeSH Terms] OR hyperalgesia[tiab] OR “neuralgia”[MeSH Terms] OR neuralgia[tiab] OR neuralgias[tiab] OR sore[tiab] OR soreness[tiab] OR ache[tiab] OR aches[tiab] OR discomfort[tiab] OR distress[tiab] OR hurt[tiab] OR hurts[tiab] OR hurting[tiab]

1. **Rodents**

“mice”[MeSH Terms] OR mice[tiab] OR mus[tiab] OR mouse[tiab] OR murine[tiab] OR murines[tiab] OR muridae[tiab] OR m. musculus[tiab] OR “rats”[MeSH Terms] OR rats[tiab] OR rat[tiab] OR rattus[tiab] OR “rodentia”[MeSH Terms] OR rodentia[tiab] OR rodent[tiab] OR rodents[tiab]

**Embase**

1. **Specific tests**

Nesting/ OR (nesting OR nest building).ti,ab,kw. OR burrowing.ti,ab,kw. OR facial expression/ OR (facial expression OR face expression OR grimace).ti,ab,kw. OR facial grimacing/ OR facial grimacing.ti,ab,kw. OR face pain/ OR ((facial AND pain) OR (pain AND face)).ti,ab,kw. OR faces pain scale/ OR ((faces AND pain AND scale) OR (faces AND pain AND scales) OR (face AND pain AND scale) OR (face AND pain AND scales)).ti,ab,kw. OR pain assessment/ OR (pain assessment OR pain assessments OR assessment of pain).ti,ab,kw. OR pain measurement/ OR (pain measurement OR pain measurements OR pain evaluation).ti,ab,kw. OR experimental pain test/ OR ((experimental AND pain AND test) OR (experimental AND pain AND tests)) .ti,ab,kw.

1. **Surgery**

Surgery/ OR (surgery OR surgeries OR surgical OR surgically OR postsurgical OR postsurgically OR postoperative OR postoperatively OR operation OR operative OR operations).ti,ab,kw. OR surgical wound/ OR incision/ OR (incision OR incisions OR incisional).ti,ab,kw. OR laparotomy/ OR (laparotomy OR laparotomies OR laparoscopy OR laparoscopies).ti,ab,kw. OR craniotomy/ OR (craniotomy OR craniotomies).ti,ab,kw. OR amputation/ OR (amputation OR amputations).ti,ab,kw. OR transplantation/ OR (transplantation OR transplantations OR graft OR grafts OR grafting).ti,ab,kw. OR implantation/ OR (implantation OR implantations).ti,ab,kw. OR hysterotomy.ti,ab,kw. OR (hysterectomy OR hysterectomies).ti,ab,kw. OR neurosurgery/ OR (neurosurgery OR neurosurgical OR neurosurgically).ti,ab,kw. OR orthopedics/ OR (orthopedic OR orthopedics OR orthopaedic OR orthopaedics).ti,ab,kw.

1. **Pain**

Pain/ OR (pain OR pains OR physical suff* OR sore OR soreness OR ache OR aches OR discomfort OR distress OR hurt OR hurts OR hurting).ti,ab,kw. OR nociception/ OR nociception.ti,ab,kw. OR allodynia/ OR allodynia.ti,ab,kw. OR hyperalgesia/ OR hyperalgesia.ti,ab,kw. OR neuralgia/ OR (neuralgia OR neuralgias).ti,ab,kw.

1. **Rodents**

Mouse/ OR (mouse OR mice).ti,ab,kw. OR mus musculus/ OR mus musculus.ti,ab,kw. OR murine/ OR (murine OR murines).ti,ab,kw. OR muridae/ OR muridae.ti,ab,kw. OR rat/ OR (rat OR rats OR rattus).ti,ab,kw. OR rodent/ OR (rodent Or rodents OR rodentia).ti,ab,kw.

**Web of science**

1. **Specific test**

TS=(nesting OR “nest building” OR burrowing OR grimace OR “facial grimacing” OR “facial expression” OR “face expression” OR facial pain OR pain face OR faces pain scale OR faces pain scales OR face pain scale OR face pain scales OR “pain assessment” OR “pain assessments” OR “assessment of pain” OR “pain measurement” OR “pain measurements” OR experimental pain test OR experimental pain tests OR “pain evaluation”)

1. **Surgery**

TS=(surgery OR surgeries OR surgical OR surgically OR postsurgical OR postsurgically OR postoperative OR postoperatively OR incision OR incisions OR incisional OR operation OR operative OR operations OR laparotomy OR laparotomies OR laparoscopy OR laparoscopies OR craniotomy OR craniotomies OR amputation amputations OR transplantation OR transplantations OR graft OR grafting OR implantations OR implantations OR hysterotomy OR [hysterectomy](https://en.wikipedia.org/wiki/Hysterectomy) OR hysterectomies OR neurosurgery OR neurosurgical OR neurosurgically OR orthopedic OR orthopaedic OR orthopedics OR orthopaedics)

1. **Pain**

TS=(Pain OR pains OR “physical suff*” OR nociception OR allodynia OR hyperalgesia OR neuralgia OR neuralgias OR sore OR soreness OR ache OR aches OR discomfort OR distress OR hurt OR hurts OR hurting)

1. **Rodents**

TS=(mice OR mus OR mouse OR murine OR murines OR muridae OR “m. musculus” OR rats OR rat OR rattus OR rodent OR rodents OR rodentia)

**Supplementary table S1: List of all included studies and evaluated parameters**

| **Study ID** | **Title** | Grimace | Nest building | Burrowing |
| --- | --- | --- | --- | --- |
| Abdelrahman_2019 | A novel multi-parametric analysis of non-invasive methods to assess animal distress during chronic pancreatitis |  |  |  |
| Akintola_2017 | The grimace scale reliably assesses chronic pain in a rodent model of trigeminal neuropathic pain | mouse + rat |  |  |
| Andrews_2011 | Spontaneous burrowing behaviour in the rat is reduced by peripheral nerve injury or inflammation associated pain |  |  |  |
| Arras_2007 | Assessment of post-laparotomy pain in laboratory mice by telemetric recording of heart rate and heart rate variability |  |  |  |
| Beninson_2018 | Analgesic efficacy and hematologic effects of robenacoxib in mice |  |  |  |
| Cesarovic_2014 | Impact of inhalation anaesthesia, surgery and analgesic treatment on home cage behaviour in laboratory mice |  |  |  |
| Chaves_2018 | Influence of tramadol on functional recovery of acute spinal cord injury in rats |  |  |  |
| Chi_2013 | Postoperative pain impairs subsequent performance on a spatial memory task via effects on N-methyl-D-aspartate receptor in aged rats |  |  |  |
| Cho_2019 | Evaluating analgesic efficacy and administration route following craniotomy in mice using the grimace scale |  |  |  |
| Clemensen_2018 | Transdermal fentanyl solution provides long-term analgesia in the hind-paw incisional model of postoperative pain in male rats |  |  |  |
| De Rantere_2016 | The relationship between the Rat Grimace Scale and mechanical hypersensitivity testing in three experimental pain models |  |  |  |
| Deseure_2018 | Orofacial neuropathic pain reduces spontaneous burrowing behavior in rats |  |  |  |
| Dwivedi_2016 | Differential expression of PCSK9 modulates infection, inflammation, and coagulation in a murine model of sepsis |  |  |  |
| Evangelista-Vaz_2018 | Analgesic Efficacy of Subcutaneous–Oral Dosage of Tramadol after Surgery in C57BL/6J Mice |  |  |  |
| Falkenberg_2019 | Clinical, Physiologic, and Behavioral Evaluation of Permanently Catheterized NMRI Mice |  |  |  |
| Faller_2015 | Refinement of analgesia following thoracotomy and experimental myocardial infarction using the Mouse Grimace Scale |  |  |  |
| Fujita_2018 | Allopregnanolone suppresses mechanical allodynia and internalization of neurokinin-1 receptors at the spinal dorsal horn in a rat postoperative pain model |  |  |  |
| Gallo_2020 | Tell-tale TINT: Does the Time to Incorporate into Nest Test Evaluate Postsurgical Pain or Welfare in Mice? |  |  |  |
| Gao_2017 | Local infiltration of the surgical wounds with levobupivacaine, dexibuprofen, and norepinephrine to reduce postoperative pain: A randomized, vehicle–controlled, and preclinical study |  |  |  |
| Georgieva_2019 | Fatty acid suppression of glial activation prevents central neuropathic pain after spinal cord injury |  |  |  |
| Guo_2017 | Thalidomide alleviates postoperative pain and spatial memory deficit in aged rats |  |  |  |
| Harikrishnan_2019 | A novel technique to develop thoracic spinal laminectomy and a methodology to assess the functionality and welfare of the contusion spinal cord injury (SCI) rat model |  |  |  |
| Herndon_2016 | Sustained-release buprenorphine improves postsurgical clinical condition but does not alter survival or cytokine levels in a murine model of polymicrobial sepsis |  |  |  |
| Hsi_2020 | Hypoglycemia after Bariatric Surgery in Mice and Optimal Dosage and Efficacy of Glucose Supplementation |  |  |  |
| Jeger_2017 | Improving animal welfare using continuous nalbuphine infusion in a long-term rat model of sepsis |  |  |  |
| Jirkof_2010 | Burrowing behavior as an indicator of post-laparotomy pain in mice |  |  |  |
| Jirkof_2012 | Individual housing of female mice: influence on postsurgical behaviour and recovery |  |  |  |
| Jirkof_2013a | Housing of female mice in a new environment and its influence on post-surgical behaviour and recovery |  |  |  |
| Jirkof_2013b | Assessment of postsurgical distress and pain in laboratory mice by nest complexity scoring |  |  |  |
| Jirkof_2015 | Buprenorphine for pain relief in mice: repeated injections vs sustained-release depot formulation |  |  |  |
| Jirkof_2018 | Tramadol: Paracetamol in drinking water for treatment of post-surgical pain in laboratory mice |  |  |  |
| Katri_2019 | A dual amylin and calcitonin receptor agonist inhibits pain behavior and reduces cartilage pathology in an osteoarthritis rat model |  |  |  |
| Kawano_2014 | Effects of ketoprofen for prevention of postoperative cognitive dysfunction in aged rats |  |  |  |
| Kawano_2017 | Effects and underlying mechanisms of endotoxemia on post-incisional pain in rats |  |  |  |
| Kawano_2018 | Involvement of acute neuroinflammation in postoperative delirium-like cognitive deficits in rats |  |  |  |
| Kendall_2016 | Efficacy of sustained-release buprenorphine in an experimental laparotomy model in female mice |  |  |  |
| Klune_2019 | Comparing the Rat Grimace Scale and a composite behaviour score in rats |  |  |  |
| Korat_2017 | Local infiltration of the surgical wound with levobupivacaine, ibuprofen, and epinephrine in postoperative pain: An experimental study |  |  |  |
| Korat_2018 | Analgesic Effect of Local Infiltration of the Surgical Wound Containing Levobupivacaine, Ibuprofen, and Epinephrine in Rats Undergoing Laparotomy |  |  |  |
| Koyama_2019 | Acute postoperative pain exacerbates neuroinflammation and related delirium-like cognitive dysfunction in rats |  |  |  |
| Kumstel_2019 | Benefits of non-invasive methods compared to telemetry for distress analysis in a murine model of pancreatic cancer |  |  |  |
| Langford_2010 | Coding of facial expressions of pain in the laboratory mouse |  |  |  |
| Lau_2013 | A back translation of pregabalin and carbamazepine against evoked and non-evoked endpoints in the rat spared nerve injury model of neuropathic pain |  |  |  |
| Leach_2012 | The assessment of post-vasectomy pain in mice using behaviour and the Mouse Grimace Scale |  |  |  |
| Locatelli_2018 | Resveratrol-loaded nanoemulsion prevents cognitive decline after abdominal surgery in aged rats |  |  |  |
| Mai_2018 | Body temperature and mouse scoring systems as surrogate markers of death in cecal ligation and puncture sepsis |  |  |  |
| Matsumiya_2012 | Using the Mouse Grimace Scale to reevaluate the efficacy of postoperative analgesics in laboratory mice. |  |  |  |
| Miller_2016 | Using the mouse grimace scale and behaviour to assess pain in CBA mice following vasectomy |  |  |  |
| Möller_2018 | Toward evidence‐based severity assessment in rat models with repeated seizures |  |  |  |
| Muralidharan_2016 | Comparison of burrowing and stimuli-evoked pain behaviors as end-points in rat models of inflammatory pain and peripheral neuropathic pain |  |  |  |
| Nunamaker_2018 | Evaluation of analgesic efficacy of meloxicam and 2 formulations of buprenorphine after laparotomy in female Sprague–Dawley rats |  |  |  |
| Oliver_2014 | Psychometric assessment of the Rat Grimace Scale and development of an analgesic intervention score |  |  |  |
| Oliver_2018 | Using cageside measures to evaluate analgesic efficacy in mice (Mus musculus) after surgery |  |  |  |
| Pham_2010 | Housing environment influences the need for pain relief during post-operative recovery in mice |  |  |  |
| Philips_2016 | Use of the rat grimace scale to evaluate neuropathic pain in a model of cervical radiculopathy |  |  |  |
| Prefontaine_2014 | Postoperative pain in Sprague Dawley rats after liver biopsy by laparotomy versus laparoscopy |  |  |  |
| Redaelli_2019 | A refinement approach in a mouse model of rehabilitation research. Analgesia strategy, reduction approach and infrared thermography in spinal cord injury |  |  |  |
| Robinson-Junker_2019 | Sleeping through anything: The effects of unpredictable disruptions on mouse sleep, healing, and affect |  |  |  |
| Rock_2015 | The time-to-integrate-to-nest test as an indicator of wellbeing in laboratory mice. |  |  |  |
| Roughan_2016 | Meloxicam prevents COX‐2‐mediated post‐surgical inflammation but not pain following laparotomy in mice |  |  |  |
| Saine_2016 | Effects of fentanyl on pain and motor behaviors following a collagenase-induced intracerebral hemorrhage in rats |  |  |  |
| Sauer_2016 | Buprenorphine via drinking water and combined oral-injection protocols for pain relief in mice |  |  |  |
| Schneider_2017 | Application of the rat grimace scale as a marker of supraspinal pain sensation after cervical spinal cord injury |  |  |  |
| Shepherd_2018 | Deficits in burrowing behaviors are associated with mouse models of neuropathic but not inflammatory pain or migraine. |  |  |  |
| Shi_2018 | Development of an in vivo mouse model of discogenic low back pain |  |  |  |
| Sotocinal_2011 | The Rat Grimace Scale: a partially automated method for quantifying pain in the laboratory rat via facial expressions |  |  |  |
| Staib-Lasarzik_2019 | Analgesic treatment limits surrogate parameters for early stress and pain response after experimental subarachnoid hemorrhage |  |  |  |
| Thomas_2016 | Efficacy of intrathecal morphine in a model of surgical pain in rats |  |  |  |
| Tuttle_2018 | A deep neural network to assess spontaneous pain from mouse facial expressions |  |  |  |
| Van-Loo_2007 | Impact of'living apart together'on postoperative recovery of mice compared with social and individual housing |  |  |  |
| Waite_2015 | Efficacy of common analgesics for postsurgical pain in rats |  |  |  |
| Yamanka_2017 | The preventive effects of dexmedetomidine on endotoxin-induced exacerbated post-incisional pain in rats |  |  |  |
| Yousef_2015 | Successful reconstruction of nerve defects using distraction neurogenesis with a new experimental device |  |  |  |
| Yuan_2018 | Nest‐building activity as a reproducible and long‐term stroke deficit test in a mouse model of stroke |  |  |  |
